# IXth International Symposium on Thysanoptera and Tospoviruses: 31^st^ August – 4^th^ September 2009, Sea World Resort, Queensland, Australia

**DOI:** 10.1673/031.010.14126

**Published:** 2010-10-01

**Authors:** Denis Persley, Calum Wilson, John Thomas, Murray Sharman, Desley Tree

**Affiliations:** ^1^ Department of Employment, Economic Development and Innovation, 80 Meiers Road, Indooroopilly, Queensland 4068.; ^2^ Tasmanian Institute of Agricultural Research, University of Tasmania, New Town Research Laboratories, 13 St Johns Avenue, New Town, Tasmania 7008, Australia.

Diseases caused by tospoviruses have become a major threat to a broad range of agricultural and horticultural crops. To date, seventeen different tospoviruses have been characterised and twelve thrips species have been identified as vectors of these viruses. Management of diseases caused by tospoviruses has become a challenge for sustainable production of vegetables in smallholder farming systems of South and Southeast Asia due to the broad host range of thrips and tospoviruses, overlapping cropping practices, indiscriminate use of insecticides resulting in vector thrips developing insecticide resistance. The Integrated Pest Management-Collaborative Research and Support Program (IPM CRSP) funded by USAID has initiated multi-disciplinary, system-wide research and technology transfer programs for a comprehensive development strategy to mitigate the impact of tospovirus diseases in smallholder agriculture in the region. Current research has focused on India and Indonesia. Tospoviruses present in India include *Peanut bud necrosis virus* in vegetables and legumes, Capsicum chlorosis virus in tomatoes and chilli peppers, Watermelon bud necrosis virus in melons, and *Iris yellow spot virus* in onions. Tospoviruses identified in Indonesia include *Tomato spotted wilt virus* in tomatoes and chilli peppers, and *Peanut bud necrosis virus* in peanuts. Major thrips species identified in India from tomatoes, chilli peppers and onions include *Thrips palmi, T. tabaci, Frankliniella schultzei, Scirtothrips dorsalis* and *T. hawaiiensis*. The first four thrips species are known vectors of tospoviruses. Although *T. palmi* is native to Indonesia, the status of other vector thrips species in the country is not yet clear. Diagnostic methods for the accurate detection of these viruses in vegetable crops have been developed. The project has also contributed to institutional capacity building within developing countries for conducting research on tospovirus diseases through graduate education and short- and medium-term training programs.

## References

[bibr01] McMichael L, Persley DM, Thomas J (2002). A new tospovirus serogroup IV species infecting capsicum and tomato in Queensland Australia.. *Australasian Plant Pathology*.

