# Supplementary material for: IXth International Symposium on Thysanoptera and Tospoviruses: 31st August – 4th September 2009, Sea World Resort, Queensland, Australia
Source: J Insect Sci. 2010 Oct 1;10:166. doi: 10.1673/031.010.14126 (PMC3388970; doi:10.1673/031.010.14126)
Supplement: Related texts

# Research article: *Heliothrips sylvanus* Faure (Thysanoptera: Thripidae) confirmed as a potential economic pest of sweet and astringent persimmon in South Africa

**Published:** 2010-10-01

**Authors:** Elleunorah Allsopp, Muriel C. Knipe

**Affiliations:** ARC Infruitec-Nietvoorbij (Fruit, Vine & Wine Institute of the Agricultural Research Council), Private Bag X5026, Stellenbosch, 7599 South Africa

*Heliothrips sylvanus* Faure is native to southern Africa and has been listed as a pest on guavas, hence the common name of guava thrips. Injury to guavas is mostly limited to scarring of leaves, although occasional scarring of fruit has been reported. *H. sylvanus* also occurs on table grapes in the Western Cape Province. It apparently prefers older, mature vine leaves and although it causes extensive feeding damage to these leaves, no damage to grape berries has been recorded. Because *H. sylvanus* populations peak towards the end of summer after most table grapes have been harvested, it is not considered to be of economic importance. In May 2007 severe insect damage to astringent persimmons (var. Triumph, also known as Sharon fruit) was reported from a farm in the Hex River Valley, Western Cape Province. Thrips collected from this persimmon orchard and adjacent table grape vines were identified as *H. sylvanus*. Feeding damage on the fruit could, however, not be conclusively ascribed to *H. sylvanus*. Groups of 10 immature and adult *H. sylvanus* from the affected persimmon orchard were confined on either green or ripening undamaged fruit of both astringent (var. Triumph) and sweet persimmon (var. Fuyugaki) in individual cages in an insectary. After five days, feeding damage by immature and adult *H. sylvanus* to green and ripening fruit of both astringent and sweet persimmons was clearly visible with the naked eye under the sepals and around the bottom ends of the fruit where they rested on the cage bottom. Examination under a stereo microscope confirmed that the thrips were feeding in these areas. These findings confirm that feeding by *H. sylvanus* can cause economic damage to persimmon fruit and that it should be regarded as a potential economic pest of persimmon.

# Research article: A serological assay for the detection of the nonstructural protein (NSs) of *Iris yellow spot virus* and its use in virus detection in plant and thrips vectors

**Published:** 2010-10-01

**Authors:** S. Bag, K.L. Druffel, S. Rondon, D.G. Riley, Hanu R. Pappu

**Affiliations:** ^1^ Department of Plant Pathology, PO Box 646430, Washington State University, Pullman, WA 99164, USA; ^2^ Hermiston Agricultural Research and Extension Center, Oregon State University, Hermiston, OR 97838; ^3^ University of Georgia, Tifton, GA 31793, USA.

*Iris yellow spot virus* (IYSV), transmitted by onion thrips *Thrips tabaci*, is one of the most economically important constraints to onion production in United States and is one of the three tospoviruses to occur in the United States. In the US, as well as on a world-wide basis, an increasing incidence of IYSV has been reported in recent years. Besides thrips, there is no evidence of virus transmission through seed. Hence, infected plants and viruliferous thrips are the primary source and means of virus spread. There are limited options available for managing IYSV outbreaks. The ability to rapidly and accurately detect IYSV in thrips vectors for the purpose of estimation of the proportion of viruliferous thrips (=transmitters) from the field could potentially provide information that would be useful in more effective thrips management practices. A polyclonal antiserum was produced to the recombinant, *E. coli*-expressed nonstructural protein (NSs) coded by the small (S) RNA of IYSV. The recombinant fusion protein was obtained in the insoluble fraction, purified using a nickel column, and was used in immunization to produce a high-titred polyclonal antiserum in rabbits. When used in an antigen-coated plate ELISA, the antiserum, diluted up to 1:4,000, could detect the virus in a single adult thrips and in plants. Availability of antiserum to a non-structural protein of IYSV would be useful in epidemiological studies to better understand the role of thrips vectors in outbreaks of this important virus of onion.

# Research article: Completion of the molecular characterisation of *Iris yellow spot virus* (IYSV) genome: Structure and genome organisation of the large RNA of IYSV

**Published:** 2010-10-01

**Authors:** S. Bag, S. Bag, K.L. Druffel, Hanu R. Pappu

**Affiliations:** Department of Plant Pathology, PO Box 646430, Washington State University, Pullman, WA 99164, USA

The structure and organisation of the large (L) RNA of *Iris yellow spot virus* (IYSV) was determined, and with this, the complete genomic sequence of IYSV has been elucidated. The L RNA was 8880 nucleotides in length and contained a single open reading frame (ORF) in the viral complementary (vc) strand. The primary translation product of 331.17 kDa shared many of the features of the viral RNA-dependent RNA polymerase (RdRp) coded by L RNAs of known tospoviruses. The 5′ and 3′ termini of IYSV L RNA (vc) contain two untranslated regions of 33 and 226 nucleotides, respectively, and both termini have conserved terminal nucleotides, another common feature of tospovirus genomic RNAs. Conserved motifs characteristic of RdRps of members of *Bunyaviridae* were present in the IYSV RdRp. These included DxxKWS (motif A); QGxxxxxSS (motif B); SDD (motif C); K (motif D); and EFxSE (motif E), Furthermore, three motifs TDF (Motif F1); KxQRTK (Motif F2) and DREIY (Motif F3) found in the RdRp of *Capsicum chlorosis virus* (CaCV) were also found in the IYSV RdRp. Phylogeny showed that the RdRp of IYSV is closer to the Eurasian group of tospoviruses: *Tomato zonate spot virus* from China, *Watermelon silver mottle virus* from Taiwan, CaCV from Thailand *Groundnut bud necrosis virus* from India, and *Melon yellow spot virus* from Japan, whereas *Tomato spotted wilt virus* and *Impatiens necrotic spot virus* formed a different cluster (American), similar to that observed with the medium and small RNAs of IYSV and other tospoviruses.

# Research article: Symptomatology of *Iris yellow spot virus* in selected indicator hosts

**Published:** 2010-10-01

**Authors:** S. Bag, Hanu R. Pappu

**Affiliations:** Department of Plant Pathology, Washington State University, Pullman, WA, 99164, USA

*Iris yellow spot virus* (IYSV) is an economically important pathogen of onion bulb and seed crops. Studies on biological characteristics of the virus have been limited due to difficulties in obtaining consistent and reproducible mechanical transmission and lack of indicator hosts. Several plant species were evaluated for their response to mechanical inoculation with IYSV. The following seedlings were used: *Arabidopsis thaliana* COL 1, *Capsicum annuum* (Serrano pepper), *Chenopodium quinoa, Datura innoxia, D. ferox, D. stramonium, Nicotiana benthamiana, N. tabacum, Solanum melongena* and *Vigna unguiculata* (Heirloom variety). Infection was verified by symptoms, ELISA and RT-PCR of inoculated, and younger, non-inoculated leaves. In *N. benthamiana*, chlorotic local lesions appeared 7 to 10 days post inoculation (DPI) which subsequently expanded leading to drying of leaves by 20–25 DPI. The virus spread systematically showing severe veinal necrosis and some stem necrosis. *D. stramonium* showed 25–30 small chlorotic local lesions initially of 2–5 mm 10 to 12 DPI. The numbers of local lesions gradually increased and spread throughout the leaves within 20–25 days, and as the lesions coalesced the leaves dried 35–40 DPI. Infection remained localised. In *V. unguiculata*, symptoms appeared as diffuse and small necrotic spots in inoculated leaves 5 to 6 DPI. Chlorotic and necrotic ring spots developed in the inoculated leaves of *C. annuum*, which gradually increased in size and *C. quinoa* produced small concentric chlorotic rings spots,. Only the inoculated leaves were positive for IYSV in ELISA and RT-PCR, and no systemic infection could be seen. *A. thaliana* COL 1, *D. innooxia, D. ferox, N. tabacum* and *S. melongena* were symptomless and were negative for IYSV when tested by DAS-ELISA. There are no reports of natural infection of *C. annuum* by IYSV although *C. annuum* is a host for several other tospoviruses. Our studies showed that *C. annum* could be experimentally infected with IYSV.

# Research article: The biological control of onion thrips in stored onions

**Published:** 2010-10-01

**Authors:** Greg J. Baker, K. Powis

**Affiliations:** Entomology Unit, South Australian Research and Development Institute, GPO Box 397, Adelaide, SA 5001, Australia

Despite intensive field spray programmes, harvested onion bulbs in storage on Australian farms are invariably infested by onion thrips, *Thrips tabaci* Lindeman. Onion thrips present in the harvested bulbs continue to reproduce and develop in storage, and onion thrips continue to fly in and colonise the onions in the open stored sheds. The thrips feeding causes tissue scarring and loosening of the outer layers of the bulb, which in turn cause the onions, particularly red, premium salad types, to be downgraded in quality and market value. No in-storage control options currently exist, in Australia or overseas. We report on a study which investigated the potential for inundative release of two commercially-available (Biological Services, Loxton, S. Aust.) predatory mite species, the phytoseiid *Neoseiulus cucumeris* (Oudemans) and the ascid *Hypoaspis aculeifer* Canestrini, to limit onion thrips damage in stored red onions. The most effective control was achieved using *N. cucumeris*. The effects of rate, method and timing of *N. cucumeris* release were assessed, and a cost-effective inundative method devised using approximately 1.6x10^3^ *N. cucumeris* per tonne of onions sprinkled in a vermiculite mix over the top of the onion bins at, or soon after, harvest.

# Research article: Conservation biological control of Kelly's citrus thrips in Australian citrus orchards using soil-dwelling predatory mites

**Published:** 2010-10-01

**Authors:** Greg J. Baker, P. Crisp

**Affiliations:** Entomology Unit, South Australian Research and Development Institute, GPO Box 397, Adelaide, SA 5001, Australia

Kelly's citrus thrips (KCT), *Pezothrips kellyanus* (B agnail), feed on citrus fruit, causing cosmetic blemish and market downgrade costing the Australian industry an estimated >$15Mpa. Current control programmes rely heavily on foliar application of chlorpyrifos and methidathion. KCT have developed tolerance to each of these organophosphates, leading to increased spray frequency and IPM disruption. In citrus, KCT is soil pupating. Arboreal and ground (surface)-foraging invertebrate predators do not appear to suppress populations of KCT to commercially-acceptable levels. However, a suite of soil-dwelling predatory mites have been identified as a potentially effective biological control; where their populations are elevated emergence of KCT from the soil is reduced by 50% or greater. Several of these mite species have been shown to survive and reproduce on KCT. The abundance of the soil-dwelling predatory mite populations is positively correlated with soil organic C levels. The potential for manipulation of these mite populations using various soil amendments, to provide greater regulation of KCT, has been assessed. Recycled green organics, grape mark and animal manure have been applied at several rates to orange trees, and KCT and beneficial mite densities measured. The results of these trials will be presented. Interestingly, these soil treatments have improved water use efficiency, yield and size of fruit, and the costs of their application are offset in the first year from increased returns independent of the thrips control benefit. Further, the toxicity of a number of citrus insecticides to this complex of soil-dwelling predatory mites has been evaluated to assess their suitability for integrated management of KCT. Foliar runoff of the organophosphates, particularly chlorpyrifos, is highly toxic and disruptive to these soil predators. A number of newer insecticides appear to be more benign and better suited to an IPM programme based on conservation of soil predators. These results will also be presented.

# Research article: Thrips as pests in pome and stonefruit in Western Australia

**Published:** 2010-10-01

**Authors:** Sonya Broughton

**Affiliations:** Western Australian Department of Agriculture, Baron-Hay Court, South Perth WA 6151, Australia.

Prior to the accidental introduction of western flower thrips (*Frankliniella occidentalis* (Pergande)) to Australia in 1993, the native plague thrips (*Thrips imaginis* Bagnall) was most commonly associated with fruit damage to stone and pomefruit. Early (apples, plums) and/or late season damage (nectarines) attributed to *F. occidentalis* has been recorded from all states where *F. occidentalis* occurs, with the exception of Tasmania. To determine which species of thrips were causing reported damage; thrips were collected and identified from infested fruit. Different species of thrips were also caged with nectarines, plums and apples to determine what type of damage they caused. Early season damage to apples was caused by *F. occidentalis* at petal fall and was characterised by the formation of ‘pansy spots’. Early season damage to plums was caused by *F. occidentalis* and *T. imaginis*, with observed damage including pansy spot, fruit scarring resembling blotches and corky spots. Late season damage to nectarines was caused primarily by *F. occidentalis*.

# Research article: Evaluation of colour and pheromones for monitoring thrips (Thysanoptera:Thripidae) in pome and stone fruit

**Published:** 2010-10-01

**Authors:** Sonya Broughton

**Affiliations:** Western Australian Department of Agriculture, Baron-Hay Court, South Perth WA 6151. Australia.

Three trap trials were conducted in commercial pome and stone fruit orchards to determine the effects of different trap base colours and a synthetic version of a *Frankliniella occidentalis* (Pergande) sexual aggregation pheromone (Thripline_ams_) on thrips catches (Thysanoptera: Thripidae). Catches of beneficial insects including syrphids (Diptera: Syrphidae), lacewings (Neuroptera: Chrysopidae, Hemerobiidae) and ladybirds (Coleoptera: Coccinellidae) were also evaluated. More thrips were attracted to blue, yellow and white sticky traps compared to green, clear, red or black traps. Blue was most attractive to beneficial insects, followed by white, red, black, yellow and green traps. Clear traps were the least attractive. Significantly higher numbers of western flower thrips (*Frankliniella occidentalis* (Pergande)) were caught on traps baited with pheromone. Based on these results, yellow traps baited with Thripline_ams_ are recommended for potential use in monitoring *F. occidentalis*.

# Research article: Effect of insecticides on *Orius armatus* Gross (Hemiptera: Anthocoridae)

**Published:** 2010-10-01

**Authors:** Sonya Broughton, L. Chilman

**Affiliations:** ^1^ Western Australian Department of Agriculture, Baron-Hay Court, South Perth WA 6151; Australia; ^2^ ManChil IPM Services, PO Box X, Muchea, WA., Australia

In Australia the native anthocorid, *Orius armatus* Gross, is being mass reared for biological control of western flower thrips (*Frankliniella occidentalis* (Pergande)). *Orius armatus* is the only *Orius* species available to growers in Australia, and feeds on larval and adult *F. occidentalis*. The first crop being targeted for *O. armatus* releases are glasshouse capsicum (peppers). To determine the compatibility of insecticides commonly used by capsicum growers with *O. armatus,* we exposed laboratory reared adults to insecticides residues applied to potted capsicum plants in the greenhouse, and filter paper in laboratory tests. Insects were exposed for 48-hours and mortality determined at 24 and 48 hours.

# Research article: *Orius armatus* - an Australian anthocorid predator of Western flower thrips (*Frankliniella occidentalis*)

**Published:** 2010-10-01

**Authors:** Sonya Broughton, L. Chilman

**Affiliations:** ^1^ Western Australian Department of Agriculture, BaronHay Court, South Perth WA 6151; ^2^ ManChil IPM Services, PO Box X, Muchea, WA. Australia

The tribe Oriini (Anthocoridae) has a worldwide distribution but is poorly represented in Australia. The largest genus is *Orius* Gross, which has six species: *Orius armatus* Gross, *O. chadwicki* Woodward & Postle, *O. heterorioides* Woodward and Postle, *Orius tantillus* (Motschulsky), *O. dendrophilus* sp. n. and *O. gracilis* sp. n. Of these, *O. armatus* is the only species commercially available to Australian growers. *O. armatus* has been collected from native and introduced flowering trees (*Acacia* spp., eucalypts) and exotic crops including carnation, corn, cotton, sorghum and sunflower. Its native prey is *Helicoverpa* spp. eggs, though adults and nymphs will also feed on aphids (*Myzus persicae, Rhopalosiphum maidis*) and larval and adult *F. occidentalis*. The selection of the right crop will be essential for the efficient use of *O. armatus* in Australia. As with other *Orius* species, *O. armatus* are likely to perform better on crops with abundant pollen such as capsicums, and in protected crops rather than field crops.

# Research article: Advancing WFT/TSWV control using inundative release of beneficials and habitat manipulation (greenhouse systems)

**Published:** 2010-10-01

**Authors:** Tony Burfield

**Affiliations:** Extension Officer, South Australian Research and Development Institute (SARDI) GPO Box 397 Adelaide SA 5001, Australia

'Western flower thrips, *Frankliniella occidentalis* (WFT) and the *Tomato spotted wilt virus* (TSWV) that they transmit is the major crop protection issue for most greenhouse growers in Virginia. Capsicums (sweet peppers) are highly susceptible to TSWV and the most vulnerable crop on the Northern Adelaide Plains (NAP) followed by lettuces both hydroponic and field. The *Tsw* gene conferring resistance to TSWV afforded capsicum growers short lived relief in the 2001-02 season, however the gene was largely overcome by a new strain of TSWV that impacted in 2002 (Reference is Australasian Plant Pathology 35, 123–128 (2006). Pest and virus control is elusive and failures are commonly attributed to pesticide resistance.

Vegetable levy funded research and extension efforts to tackle this issue have progressively shifted focus from improving pesticide practices and farm hygiene to transferring newer technologies that emphasise inundative release of biological control agents. These releases are also being complimented with habitat manipulation to replace weeds by using native plants that do not harbour pests or crop virus. In addition wild populations of a parasitic wasp (*Ceranisus* sp.) of WFT have been identified from saltbush plants in managed refuges adjacent to crops on the NAP.

This progress represents a major breakthrough for a region with a high concentration of low-tech protected cropping systems and renowned for its associated WFT and TSWV pressure. Now there is a real prospect of opening a potential (new) market for commercially reared beneficial organisms to control WFT. Further research is required to build a robust knowledge base to underpin these promising advances.

The presentation summarised encouraging IPM research and pest and disease prevention strategies that are advancing IPM practices as growers are persuaded to change key farm management and investment decisions.

# Research article: Expressed NSs protein of *Capsicum chlorosis virus* and use of its antibody for species specific diagnosis

**Published:** 2010-10-01

**Authors:** Pissawan Chiemsombat, Surasak Khankhum, Ratchanee Hongprayoon

**Affiliations:** ^1^ Department of Plant Pathology, Faculty of Agriculture at Khamphaeng Saen, Kasetsart University Khamphaeng Saen Campus, Nakhonpathom Province, 73140 Thailand; ^2^ Department of Biology Faculty of Science Mahasarakham University Khamrieng Kantarawichai District Mahasarakham Province, 44150, Thailand

In Thailand *Capsicum chlorosis tospovirus* (CaCV) causes severe damage to many important crops such as peanut, pepper and tomato. The detection of CaCV by ELISA is routinely performed using tospovirus serogroup IV antibody, but virus species could not be determined. This study aimed to develop a detection tool for specific CaCV diagnosis by using antibody against viral non structural (NSs) protein. NSs gene fragment of 1320 nt was amplified by RT-PCR from CaCV infected peanut tissues (CaCV-PKK), cloned and sequenced. The NSs-PKK gene coded for 439 amino acid residues and showed about 85–90 % identities to NSs proteins of CaCV-AIT from Thailand CaCV-CP isolate from China and *Gloxinia tospovirus. In vitro* expression of NSs protein yielded approximately 2.98 mg/ml of 50 kDa polypeptides and the protein was subsequently used as immunogen for producing polyclonal antibody in a rabbit. Obtained CaCV-NSs antiserum has titre at approx. 6×10^4^ against 3 µg of the purified 6XHis-NSs protein by ELISA. The dilution endpoint when tested with crude sap of CaCV infected peanut was 10^-3^ in phosphate buffer. This NSs antiserum reacted specifically to CaCV in infected peanut, pepper and tomato, while it did not react to plants infected by serogroup IV-*Watermelon silver mottle virus* (WSMoV) or *Melon yellow spot virus* (MYSV). In addition, the antibody against tospovirus group IV did not react with the expressed NSs-PKK protein. Our results indicated that antibody against NSs protein of CaCV is useful for specific CaCV diagnosis.

# Research article: Molecular characterisation of new tospoviruses: abundant accumulation of a dimer of the S segment during multiplication of the tospovirus *Polygonum ringspot virus*

**Published:** 2010-10-01

**Authors:** M. Ciuffo, Massimo Turina

**Affiliations:** Istituto di Virologia Vegetale, Sez. di Torino, CNR, Strada delle Cacce n73, Torino 10135, Italy

We have recently reported the isolation of two new tospovirus species (Ciuffo et al., 2008; Ciuffo et al., 2009). Melon severe mosaic virus (MeSMV) infects cucurbit crops and has typical tospovirus features. In Mexico it is widespread and causes severe disease in important agricultural areas. Another recently characterised virus, *Polygonum ringspot virus* (PolRSV), was isolated from a common weed (*Polygonum convolvulus*) in Italy. This new tospovirus is widespread, and has a wide experimental host range, but it has not yet entered economically important crops because its vector, a newly discovered thrips vectoring tospoviruses, does not feed on these. PolRSV has some interesting features that separate it from other tospoviruses. The intergenic sequence of the small (S) genome segment is unusually short, and its predicted secondary structure does not display the hairpin structure typical of other tospoviruses. Lack of this hairpin would require a mechanism for generating the subgenomic messenger RNA different from that of other tospoviruses. A second surprise is that in Northern blots of RNA extracts from *N. benthamiana* plants, the most abundant RNA species hybridizing with different S segment-derived probes is a band of circa 4.5 kb. This RNA is not present in purified virus from the same *N benthamiana* plants, where the expected S genome segment is instead present. The sequence of the ‘aberrant’ RNA showed that it is composed of a full length S segment joined covalently to a second S segment that lacks the first 300 bases at its 5′ end. We developed a qRT-PCR assay using a specific Taqman probe for this dimer. We show that it accumulates in various solanaceous experimental hosts, although we could not demonstrate its accumulation in infected thrips.

# Research article: The use of molecular technology for quarantine thrips identification: brave new world?

**Published:** 2010-10-01

**Authors:** Dom W. Collins, R. Glover, N. Boonham

**Affiliations:** The Food and Environment Research Agency, Sand Hutton, York, Y041 1LZ, UK

The ongoing development of molecular technology offers tools of ever-increasing speed and scope that can be utilised by quarantine diagnostic laboratories. There is current interest in moving from indirect assays such as real-time PCR, often targeted at specific taxa, to direct sequencing techniques (DNA barcoding). This offers a broader approach, continually building up a reference database into which an increasing number of species may be inserted, which is attractive not least when the logistical possibilities being opened up with next generation sequencing are also considered. Laboratory work has suggested that the Cytochrome oxidase I (COI) gene is a suitable locus for discriminating between species of Thrips and for other Thripidae. Work to build up a reference database of thrips species is on-going, primarily from UK field-collected populations and material from import inspections. Blind tests on the extracted DNA using the program MOTU-define produced close, but not perfect, correlation between morphological and molecular determination of segregates at the specific level, emphasising a continuing role for taxonomic interpretation. For example, suggestions from COI data of a possible cryptic species within *Thrips palmi* were supported neither by data from other loci, nor by contextual consideration of the source material. By contrast, two long-established species, *Thrips fuscipennis* and *T. sambuci*, could not be separated using COI data, albeit with limited material. For successful species discrimination, segregates based on ‘barcode gaps’, or the absences thereof, need to relate to groupings of real biological significance, but may themselves be defined by insufficient sampling of species and populations, or other factors. Conversely, they may simply disagree with an incorrect historical taxonomy. For a quarantine diagnostician DNA-sequencing, used carefully and in conjunction with morphological and biological analysis, offers the possibility of a significant increase in species resolution across all life stages and an ever-increasing range of taxa.

# Research article: Too hot to smell? Effect of temperature on response of *Frankliniella occidentalis* to chemical lures

**Published:** 2010-10-01

**Authors:** Melanie Davidson, R. Butler, C. Sansom, N. Perry, D. Teulon

**Affiliations:** Plant & Food Research, Private Bag 4704, Lincoln, Christchurch, New Zealand

We have indications from the use of thrips chemical lures in commercial greenhouses that these work less well at higher temperatures. Therefore, we examined the effect of temperature (25 and 35 °C) on the walking response of starved *Frankliniella occidentalis* (Western flower thrips WFT) females toward known thrips chemical lures (*p*-anisaldehyde, methyl isonicotinate) using a Y-tube olfactometer. At 25 °C, significantly more thrips walked up the odour-laden arm (i.e. attractive) towards *p*-anisaldehyde at doses of 0.1 and 5 µl (73, 65% respectively), but not at the other doses tested (1, 25, 125 µl). At 35 °C *p*-anisaldehyde was not attractive at any of the doses tested. At 25 °C methyl isonicotinate was significantly attractive to WFT at doses of 0.1 to 625 µl, whereas at 35 °C only 0.1 µl was attractive. The results are discussed in relation to the potential influence of temperature on the volitle compound and the thrips

# Research article: Chemical ecology in social evolution against nest parasitism: *Kladothrips* vs. *Koptothrips*

**Published:** 2010-10-01

**Authors:** Monica De Facci, T.W. Chapman, O. Anderbrant

**Affiliations:** ^1^ Lund University, Sölvegatan 37, SE-223 62 Lund, Sweden; ^2^ Memorial University, St. John's, A1B 3X9 Newfoundland Canada

In all insect societies, semiochemicals play a crucial role for both intra-nest communication and the ability of parasites to invade their host. Our project aims to reveal what chemical cues are involved in the evolution of eusociality for defense against nest parasitism by analysing the interactions between two tubuliferan genera: the model social insect clade *Kladothrips* and its kleptoparasitic genus *Koptothrips*.

Australian gall-inducing thrips (genus *Kladothrips*) produce their domicile in developing phyllodes of wattles (*Acacia* trees). Seven of the 23 described *Kl*. species present two morphs: the macropterous reproductive individuals, and the micropterous soldiers with enlarged forelegs specialized for defence against e.g. *Koptothrips*. Depending on the species involved, the relationships between them can vary from coexistence to a deadly fighting. Interestingly, the soldiers can show deficiency in their nest-mate recognition ability possibly due to *Koptothrips* being able to camouflage themselves, appearing ‘chemically insignificant’ to their victims. Such a phenomenon most commonly evolves when host and parasite are closely related, e.g. in ants. Here, however, the interacting species do not seem to be sister taxa.

While the species have been investigated from biological and phylogenetic points of view, an integrated, interdisciplinary understanding of the chemical, physiological and evolutionary principles that have shaped chemical communication within this system is still lacking. For this reason, we intend to investigate different aspects: from assessing if the plant volatiles are important for host finding and acceptance of the nest-invaders to the identification of a probable alarm pheromone in *Kl*. anal droplets; from the detection of differences and similarities in the cuticular profiles between gall-inducers and their invaders to the comparison with solitary *Kl*. species.

# Research article: Commercialisation of thrips semiochemicals

**Published:** 2010-10-01

**Authors:** William Jan De Kogel, F.C. Griepink, R.W.H.M. van Tol, D.A.J. Teulon

**Affiliations:** ^1^ Wageningen UR, Plant Research International B. V., P.O. Box 69, 6700 AB Wageningen, The Netherlands; ^2^ Plant & Food Research, Private Bag 4704, Christchurch 8140, New Zealand

Studies on semiochemicals used by thrips help us to better understand the behaviour of these insects. Semiochemicals may be related to sexual behaviour, aggregation behaviour and defense (pheromones, allomones) or host finding (kairomones). Understanding the role of semiochemicals in relation to the behaviour of thrips may also lead to the development of techniques to manipulate their behaviour. The end products of such studies are practical tools for growers to help them protect their crops from thrips infestation. These tools include both monitoring and control strategies. We will give an overview of the development and use of commercially available thrips semiochemicals and discuss future research directions.

# Research article: A Non-Structural Protein (NSs) of *Tomato spotted wilt virus* increases baculovirus replication efficiency in permissive and semi-permissive insect cells and larvae (Lepidoptera)

**Published:** 2010-10-01

**Authors:** V.C. De Oliveira, L. Bartasson, B.M. Ribeiro, Renato Resende

**Affiliations:** Dept. of Cell Biology, University of Brasilia, PO Box 70910-900, Brasilia, DF, Brazil

The NSs protein of the tospovirus *Tomato spotted wilt virus* (TSWV) has been identified as a RNAi suppressor in plant cells. In the present study, a recombinant baculovirus (vAcNSs) containing the NSs gene under the control of the viral polyhedrin (polh) gene promoter, was constructed in order to assess the effect of heterologous expression of the NSs protein on baculovirus replication in insect cells and insect larvae. We have demonstrated that permissive *Trichoplusia ni* (BTI-Tn5B1-4) and semi-permissive *Anticarsia gemmatalis* (UFL-AG-286) cell lines, infected with vAcNSs, produced more significant amounts of budded virus (BVs), when compared with wild type baculovirus. Cells and larvae were infected with a recombinant virus containing the green fluorescent protein gene (*gfp*) vHSGFP alone and co-infected with vAcNSs and vHSGFP. Co-infection with vAcNSs and vHSGFP strongly increased GFP expression in BTI-Tn5B1-4 and UFL-AG-286 cells. *A. gemmatalis* larvae infected with both viruses showed increasing population of infected hemocytes up to 72 h.p.i. in contrast with larvae infected only with vHSGFP which showed decreasing populations of infected hemocytes from 24 h.p.i. onwards. For intrahaemocoelic infection, it was obtained LD50 values of 7.15 and 1.18 PFU/larvae for AcMNPV and vAcNSs, respectively. The mechanisms involved in the increased replication of the recombinant virus containing the NSs gene in insect cells and insects are currently under investigation. Financial support: UnB, CNPq, FAP-DF

# Research article: Development of a locus-specific, co-dominant SCAR marker for assisted-selection of the *Sw*-5 (*Tospovirus* resistance) gene cluster in a wide range of tomato accessions

**Published:** 2010-10-01

**Authors:** E.C. Dianese, M.E.N. Fonseca, R. Goldbach, R. Kormelink, A.K. Inoue-Nagata, L.S. Boiteux, Renato O. Resende

**Affiliations:** ^1^ Laboratory of Phytopathology/IB/UnB, 70910-900, DF, Brazil; ^2^ CNPH/Embrapa Vegetables, CP 218, 70359-970, DF, Brazil; ^3^ Binnenhaven 11, 6709 PD, Wageningen, The Netherlands

The best levels of broad-spectrum *Tospovirus* resistance reported in tomatoes thus far are conferred by the *Sw-5* locus. This locus contains at least five paralogues (denoted *Sw-5a* through *Sw-5e*), of which *Sw-5b* represents the actual resistance gene. Here we evaluated a panel of seven PCR primer pairs matching different sequences within a genomic region spanning the *Sw-5a* and *Sw-5b* gene cluster. Primer efficiency evaluation was done employing tomato isolines with and without the *Sw-5* locus. One primer pair produced a single and co-dominant polymorphism between susceptible and resistant isolines. Sequence analysis of these amplicons indicated that they were specific for the *Sw-5* locus and their differences were due to insertions/deletions. The polymorphic SCAR amplicon encompass a conserved sequence of the promoter region of the functional *Sw-5b* gene, being located in the position -31 from its open reading frame. This primer pair was also evaluated in field assays and with a collection of accessions known to be either susceptible or resistant to tospoviruses. An almost complete correlation was found between resistance under greenhouse/field conditions and the presence of the marker. Therefore, this primer pair is a very useful tool in marker-assisted selection systems in a large range of tomato accessions.

Financial support: UnB, CNPq, CAPES, FAP-DF, Embrapa.

# Research article: Survey of thrips and *Tomato spotted wilt virus* incidence in vegetable fields and adjacent weeds in Georgia, USA

**Published:** 2010-10-01

**Authors:** Stan Diffie, David G. Riley

**Affiliations:** Department of Entomology, University of Georgia, Tifton, GA 31793, USA

*Tomato spotted wilt virus* (TSWV) (family *Bunyaviridae*; genus *Tospovirus*) causes significant yield loss annually in Georgia (USA) vegetable fields. The two main vectors of TSWV in Georgia are the tobacco thrips *Frankliniella fusca* (Hinds) and the western flower thrips *F. occidentalis* (Pergande). Weeds surrounding tomato and pepper fields in four South Georgia counties were collected monthly (2004–06) and bi-monthly (2007–08). Berlese funnels were used to extract thrips from the samples into 60% ETOH, and ELISA was run on sub-samples to test for TSWV. Yellow sticky cards were placed in the fields and on the borders of the fields for a one week period on each sampling date. In Berlese samples, immatures comprised the largest number of thrips collected (77% of the total). Suspected non-vector thrips adults comprised 12% of the total followed by *F. occidentalis* (7%) and *F. fusca* (3%). *Frankliniella fusca* was the dominant vector collected during the spring and summer while *F. occidentalis* was dominant in the fall and winter months. The number of thrips collected on sticky cards remained high throughout the warm months but had a drastic reduction during the winter and early spring months. More thrips were collected in the weeds surrounding the crop than in the crop itself. The average number of thrips collected on cards located in the area adjacent to the field was 61.3±3.32 [n=1521] compared to 30.9±1.65 [n=1507] in the field. The percentage of TSWV in the weeds adjacent to the fields ranged from 10.0%±0.04 [n=180] to 24.7%±0.03 [n=631] in 2007 and 2005, respectively. In the crops, the lowest incidence of TSWV was in Spring 2007 (0.9%) and the highest was in Spring 2005 (9.8%).

# Research article: Natural host ranges of *Tomato zonate spot virus* in Yunnan

**Published:** 2010-10-01

**Authors:** J-H. Dong, Z-K. Zhang, Y-Y. Yin, X-F. Cheng, M. Ding, Q. Fang

**Affiliations:** ^1^ Biotechnology and Genetic Germplasm Institute, Yunnan Academy of Agricultural Sciences, Kunming 650223, China; ^2^ Key Laboratory of Agricultural Biotechnology of Yunnan Province, Kunming 650223, China

*Tomato zonate spot virus* (TZSV) has become a major constraint for numerous horticultural and ornamental crops. In 2008, due to the tomato diseases caused by TZSV, 1000 kilogram of diseased tomatoes sold for just 10 Yuan (RMB). A two-year survey was conducted from Aug. 2006 to Oct. 2008 in Kunming to determine the plant host-ranges of TZSV. A total of 481 samples were collected, and tested using the polyclonal antibodies to the nucleocapsid protein (NP) of TZSV expressed in *Escherichia coli*. The testing results showed that tomato, chili pepper, tobacco, potato, spinach, lettuce, radish, parsley, endivesprout, taro, kidney bean, and various species of weeds including *Rumex dentatus, Chenopodium serotinum, Bidens pilosa, Capsella bursapastoris, Comnyza canadensis, Malachium aquaticum, Pharbitis purpurea, C. Amaranticolor, Plantago asiatica, Oenothera erythrosepala, Taraxacum officnala, Lactuca sativa* L, *Galinsoga parviflora* were natural host of TZSV. Two plant species, Stramonium (*Dature stramonium* L.) and morelberry (*Physalis alkekengi* L.), were not tested positive for TZSV during the whole survey period. Kunming is called as City of Eternal Spring, where there are favorable conditions for the overwinter of these plant species that could serve as TZSV reservoirs and thrips as TZSV vectors. Further work is necessary to control the diseases caused by TZSV.

# Research article: When cereal pests migrate to nests

**Published:** 2010-10-01

**Authors:** Peter Fedor, I. Hammersteinová, M. Doričová, W. Sierka

**Affiliations:** ^1^ Comenius University, Bratislava, Slovakia; ^2^ University of Silesia, Katowice, Poland

Barley thrips, *Limothrips denticornis*, HALIDAY, 1836 (Thysanoptera: Thripidae), a grass-dwelling Holarctic element with an ability to infiltrate a wide spectrum of habitats, represents a model example to study transport mechanisms of Thysanoptera into nests. In samples from more than 1,500 nests, collected in the period of 1975–2006, a barley thrips was recorded at 17 bird and 3 mammal species, predominantly in nests built of grass and feathers, such as those inhabited by *Turdus viscivorus, T. pilaris, Lantus collurio, Acrocephalus arundinaceus, Nucifraga caryocatactes, Passer montanus, Carduelis chloris* or *Micromys minutus*. Divisive hierarchical clustering technique (DIANA) was chosen with Gower's General Similarity Coefficient (metric) to measure proximity of mixed data types (material of nests). Fourteen material categories were selected for detailed statistical analysis. In the multi-factor analysis of variance both cases Friedman chi-squared statistic and p-value show strong dependence of presence of a barley thrips on certain material. Considering the grass-dwelling way of life this may indicate that *L. denticornis* predominantly invades nidicolous synusia through phoresy on grass during nest building and the alternatives, active flight and canopy dwelling, phoresy on hosts, and floating in atmosphere are less important. Apart from the material the shape, height, size or location of a nest was not found the significant determinant for distribution of barley thrips in nidicolous synusia, thus the role of thigmotaxy to invade sheltered places was not proved statistically.

The project was financially supported by VEGA 1/4339/07 and KEGA 3/7454/09.

# Research article: Artificial intelligence — a challenge for semiautomatic thrips identification

**Published:** 2010-10-01

**Authors:** Peter Fedor, J. Vaňhara, J. Havel, O. Majzlan

**Affiliations:** ^1^ Comenius University, Bratislava, Slovakia; ^2^ Masaryk University, Brno, Czech Republic

We tested the reliability of artificial intelligence methods for prompt and effective identification and monitoring of economically important European thrips (Thysanoptera). 101 species, including European and Mediterranean Plant Protection Organization quarantine-listed and tospovirus-transmitting pests, such as *Thrips palmi* or *Frankliniella occidentalis*, were evaluated to develop a precise and reliable digital instrument for identification. For instance, 17 quantitative morphometric characters (measured as linear distances on digital images), such as head, clavus, wing, ovipositor length and width, two qualitative two-state characters (presence/absence) and sex (Fedor et al., 2008 in *Bulletin of Entomological Research*) formed the input variable computation set for cereal damaging and grain damage causing thrips of the *Limothrips* genus. Experiments with several artificial neural network types (radial basis function, linear, probabilistic and multilayer perceptrons networks) suggested multilayer perceptrons as efficient for such a purpose generally. Its architecture was constructed conventionally with three or more feed-forward layers, i.e. input, output and one or several hidden layers (Fedor et al., 2009 in *Systematic Entomology*).

Reliable species distinction would not be possible if a single character only was considered. However, in appropriate combinations (relative values), character states can be unique and specific. In a large database, lower subsystems can be established and analysed separately (e.g. *Panchaetothripinae, Frankliniella* spp., *Thrips* spp.), while also shaping a complex supersystem with high identification reliability.

A wide character range even enables the identification of damaged insects where traditional dichotomous keys are useless. In combination with semi-automated digital image-encoding software and determination according to the most reliable characters, the method may find a wide practical application in phytosanitary work.

The project was financially supported by VEGA 1/4339/07, KEGA 3/7454/09 and MSM 0021622416.

# Research article: Economic analysis of managing thrips and *Tomato spotted wilt virus* in tomato in Georgia, United States using enterprise budget

**Published:** 2010-10-01

**Authors:** Esendugue Greg Fonsah, David G. Riley, Stormy Sparks, David Langston

**Affiliations:** Associate Professor and Extension Economist, Fruits, Vegetables and Pecans, Department of Agricultural & Applied Economics, University of Georgia, Tifton, GA 31793, USA

Recent trend depicts that tomatoes and tomato products rank as the second most important vegetable crop in the United States after potatoes and potato products, contributing 20 percent of total vegetable production (Lucier and Dettmann, 2008). Moreover, tomatoes are equally ranked second in the United States in terms of production value, generating $1.3 billion after head lettuce which contributed $1.4 billion in the same time period. In 2006, 422,000 acres of tomatoes were planted in the United States. Tomato is an important economic crop in the state of Georgia. In 2008, it ranked 14^th^ in the Georgia vegetable acreage as 3,985 acres were planted. It also ranked 6^th^ in terms of farm gate value in the same time period generating $51.2 million (Boatright and McKissick, 2008). Thrips-vectored *Tomato spotted wilt virus* (TSWV) is a serious disease capable of causing damages to the plant, fruits, quality and reducing yields drastically (Riley and Pappu, 2004). Managing TSWV can be complex. For instance, metalised UV-mulch may significantly reduce TSWV, but delay tomato maturity, potentially affecting price and market window. Also, resistant tomato lines may eliminate damage due to TSWV, but could have negative horticultural attributes that standard TSWV-susceptible hybrids do not. TSWV can induce irregular ripening in fruit after packing, affecting post-harvest costs. This study is aimed at providing the optimal return per unit of enterprise using estimates of the combination of available inputs used in the various management strategies. Thereafter, the result of the differentially developed techniques and risk-rated budgets will be used to determine which of the risk-rated thrips, TSWV and IPM decision criteria would provide superior pareto-optimal economic and financial benefit to tomato growers (Fonsah et al. 2006; Fonsah and Hudgins, 2007; Byrd et al., 2006; Fonsah et al. 2005; Calkins and DiPietre, 1983).

# Research article: Natural and artificial populations of *Frankliniella occidentalis*, biotic resistance and pest status

**Published:** 2010-10-01

**Authors:** Joe Funderburk, Stuart Reitz

**Affiliations:** ^1^ University of Florida, 155 Research Road, Quincy, Florida 32351, USA; ^2^ USDA-ARS-CMAVE, 6383 Mahan Drive, Tallahassee, Florida 32308, USA

In its native southwestern North America with Mediterranean and semi-arid climates, *Frankliniella occidentalis* employs an opportunistic life history strategy, with population characteristics that include polyphagy, rapid development, high reproductive potential, vagility, and a competitive breeding structure. The species, now considered cosmopolitan, is thought to have successfully invaded regions with many different climates. In central Chile, *F. occidentalis* has replaced and possibly displaced the native flower thrips as the most common thrips species and it feeds and reproduces on the vast majority of the native and introduced plants in the agro-ecosystem. Most of the plant species are low-quality hosts where populations either decline or remain stable, but they escape predation and competition from native thrips. On a few high-quality hosts that are abundant in the spring, populations in the absence of predators and competitor species build up to very high populations that later disperse due to crowding and declining plant host quality. In Florida, *F. occidentalis* is an inferior competitor to the native thrips on both cultivated and uncultivated plant hosts, and this competitive asymmetry, along with a reduced ability to avoid predation compared to the native flower thrips, has resulted in its exclusion (but not repulsion) in the agroecosystem. There are only rare opportunities in space and time for natural population buildup on plant hosts in the absence of predation and competition. However, the species is capable of exploitation of insecticide-treated crop fields where they rapidly increase to damaging pests in the absence of predation and competition. Apparently, *F. occidentalis* similarly encounters serious biotic resistance, even near complete repulsion, in many geographic regions with different climates. Observations show that populations occur in high numbers in insecticide-treated crop fields; only small populations in the form of stray individuals are encountered on the other available plant hosts.

# Research article: Soil fertility levels correlated with *Tomato spotted wilt virus* levels

**Published:** 2010-10-01

**Authors:** R.D. Gitaitis, A.S. Csinos, C. Nischwitz, Stephen W. Mullis

**Affiliations:** ^1^ Department of Plant Pathology, University of Georgia, Tifton, GA 31793-0748, USA; ^2^ Department of Plant Sciences, Division of Plant Pathology and Microbiology, University of Arizona, Tucson, AZ, 8572t-0036, USA

The disease level for any plant disease is dependent upon the degree of virulence of the pathogen, the susceptibility of the host and a favourable environment, i.e. the disease triangle. The soil environment, including nutrient levels, can interact with the disease triangle by affecting both host susceptibility and growth and development of the pathogen. Consequently, an association of soil fertility levels with *Tomato spotted wilt virus* (TSWV) severity and incidence in tobacco, *Nicotiana tabacum*, was investigated. Soil samples were collected from field plots at the University of Georgia, Bowen Farm in Tift County, GA in 2007 and 2008. In both years, samples were collected from 25 subsites per plot in four replicated plots in the center of the field. Subsamples were combined into a composite sample for each replicate. In addition, individual soil samples were sampled from below individual plants across the entire field. The sample area exhibited a disease gradient running north to south in 2007 but not in 2008. Number of samples in 2007 and 2008 were *n* = 84 and *n* = 170, respectively. In 2007, ratios of phosphorus:magnesium, magnesium:copper, copper:boron, iron:copper, zinc:boron and magnesium:zinc were significantly correlated with TSWV levels at *P* = 0.05 for both composite samples and individual samples. In 2008, ratios phosphorus: magnesium, phosphorus:copper and iron: copper were significantly correlated with TSWV levels at *P* = 0.05 for both composite samples and individual samples. Of particular interest was the interaction of iron with copper. This interaction indicated that as the proportion of iron increased relative to copper or as copper decreased in relation to iron, the less tomato spotted wilt was observed.

# Research article: Thrips species intercepted at New Zealand borders on Snow peas from the African continent

**Published:** 2010-10-01

**Authors:** Disna Gunawardana, Sherly George

**Affiliations:** Plant Health and Environment Laboratory, MAF Biosecurity New Zealand Ministry of Agriculture and Forestry, PO Box 2095, Auckland 1140, New Zealand

The Plant Health and Environment Laboratory Entomology Group, under the Ministry of Agriculture and Forestry Biosecurity New Zealand (MAFBNZ) undertakes diagnostics of invertebrates intercepted at New Zealand's border on fresh produce and provides advice on organism regulatory status to MAFBNZ Cargo Clearance. Snow peas, *Pisum sativum* are imported into New Zealand from the USA, Europe, Australia and Africa. Three African countries, Zimbabwe, Zambia and South Africa account for 87% of snow pea imports to New Zealand. The main snow pea invertebrate interceptions are thrips. Two thrips species, *Thrips palmi* and *Caliothrips fasciatus* are considered by MAFBNZ to be high impact exotic pests. Although *Thrips palmi* and *Caliothrips fasciatus* are not recorded from Zimbabwe, Zambia and South Africa, it is important to have records of what species are coming to New Zealand on this produce. This interception data contributes to a continuous pest risk analysis process. Since 2005, 206 Thysanoptera interceptions have been recorded on snow peas from Africa. While some of these records are represented by early instar thrips larvae others are exclusively eggs. Rearing of eggs and larvae through to adults was carried out for the purpose of morphological identification. Molecular techniques were used to complement some of the morphological identifications. Only three species were intercepted during the study period, *Frankliniella occidentalis, Frankliniella schultzei* and *Thrips tabaci*. All three species are tospovirus vectors and New Zealand lists *Frankliniella schultzei* as a regulated pest. Results of morphological identification of reared adults along with molecular identification will be briefly discussed.

# Research article: Imidacloprid resistance detected in Australian *Thrips tabaci* Lindeman (Thripidae, Thysanoptera)

**Published:** 2010-10-01

**Authors:** Grant Herron, Jianhua Mo

**Affiliations:** ^1^ NSW DPF EMAI, PMB 8, Camden 2570, Australia; ^2^ NSW DPI, Yanco Agricultural Institute, PMB Yanco, Yanco 2703, Australia

Onion thrips, *Thrips tabaci* Lindeman, is a major pest of Australian field onion with their control heavily reliant on a limited number of chemicals. Australian growers have previously complained of control failures and here we document resistance in *T. tabaci* for the first time in Australia against a range of insecticides including imidacloprid. The maximum difference in response detected via comparison of the most tolerant to the least tolerant field response indicated 164- and 606-fold resistance to α-cypermethrin and λ-cyhalothrin respectively. Diazinon resistance was also detected at 27-fold and dimethoate at 5.2-fold although omethoate, malathion and methidathion resistance were not detected. Interestingly, 6.4-fold imidacloprid resistance was detected in a single population that contained 2% highly imidacloprid resistant individuals capable of surviving a 2.0 g ai / L dose. This is the first record of imidacloprid resistance in *T. tabaci*.

# Research article: Control of western flower thrips (*Frankliniella occidentalis*) in lettuce and strawberry crops in Victoria, Australia

**Published:** 2010-10-01

**Authors:** Paul A. Horne, Jessica Page

**Affiliations:** IPM Technologies Pty Ftd, PO Box 560 Hurstbridge, Vic. 3099

Western flower thrips (*Frankliniella occidentalis*) has become an increasing problem in several horticultural crops in Australia including lettuce (*Lactuca sativa* L.) and strawberries (*Fragaria ananassa* Duch.). In lettuce the problem is mainly the transmission of tomato spotted wilt virus (TSWV) but in strawberries it is direct feeding damage. Field observations in commercial crops of lettuce between 2007 and 2009 and strawberries between 2008 and 2009 in Victoria, Australia indicated that *F. occidentalis* was a problem where regular insecticide applications were made. However, *F. occidentalis* populations were reduced and kept under control where far fewer insecticides were applied (either as seedling drenches or foliar sprays). We observed that a predatory thrips, *Haplothrips victoriensis* was more abundant in crops where insecticides were withdrawn and that there was also a reduction in *F. occidentalis* populations in these same crops. Laboratory feeding trials confirmed that *H. victoriensis* could feed on *F. occidentalis*. We present data showing the factors affecting control of *F. occidentalis* in Victorian lettuce and strawberry crops.

# Research article: Expression of nucleocapsid protein is lowered in challenged transgenic tomato plants carrying nucleocapsid gene of *Peanut bud necrosis virus*

**Published:** 2010-10-01

**Authors:** K. Jagadish, Akella Vani, B.J. Nirmal Kumar

**Affiliations:** Department of Biotechnology, Indian Institute of Horticultural Research, Hessarghatta Fake Post, Bangalore-560089, India

Transgenic tomato plants were generated carrying the nucleocapsid gene (n-gene) of *Peanut bud necrosis virus*. The plants were raised under controlled conditions in a glasshouse. The level of the N-protein was estimated by ELISA before and after challenging with the virus through sap inoculation. The analysis showed that the unchallenged transgenic plants had high levels of N-protein expression, while the level of N-protein in the same plant decreased drastically 48 hours after challenge. In the non-transgenic control plants, the level of the N-protein increased from nil (before challenge) to high levels 48 hrs after challenge. The transgenic plants were symptomless 3 weeks after challenging, while non-transgenic controls developed symptoms. The reduction in the N-protein may be due to the transcript of the transgene (positive sense) forming a double stranded structure with the viral genome (negative sense) resulting in degradation of the transgene transcripts probably through dicer mechanism.

# Research article: Present status of chronic and emerging tospoviruses in India

**Published:** 2010-10-01

**Authors:** Rakesh Kumar Jain, B. Mandal

**Affiliations:** Division of Plant Pathology, Indian Agricultural Research Institute, New Delhi-110012, India

Tospoviruses (Family *Bunyviridae*) are fast emerging as serious pathogens negatively impacting the cultivation of several field and horticultural crops in India. Of the 19 tospovirus species recorded worldwide, five have been reported from India: *Capsicum chlorosis virus* (CaCV) on capsicum, *Groundnut bud necrosis virus* (GBNV) on groundnut, *Iris yellow spot virus* (IYSV) on onion, *Peanut yellow spot virus* (PYSV) on groundnut and *Watermelon bud necrosis virus* (WBNV) on watermelon. CaCV, GBNV, PYSV and WBNV are of Indian or Asian origin, whereas IYSV is distributed worldwide. The earliest recorded tospovirus is GBNV, which has been known since 1949. Subsequently, PYSV and WBNV emerged on groundnut and watermelon respectively in 1990s, and IYSV and CaCV emerged recently on onion and capsicum during 2002–2006. *Thrips palmi* and *Scirtothrips dorsalis* are the predominant thrips vectoring tospoviruses in India. Of the Indian tospoviruses, GBNV and WBNV are endemic and the most widespread, causing up to 100% crop losses. Except for IYSV and PYSV, other tospovirus species are serologically indistinguishable. Complete genome properties are studied only for GBNV and PYSV. Other tospovirus species are recognised based on nucleocapsid protein (N) gene characteristics. Most of the Indian tospoviruses have been traced in several crop species other than their original hosts. Adaptation on new crop species other than the original host is an indication of emerging tospoviral problems in Indian agriculture. Whether the new tospoviruses have emerged within the ecosystem or been introduced from other countries is difficult to pinpoint at the moment. However, intensive cultivation, introduction of susceptible genotypes, favourable environmental conditions for thrips populations, change in genetic makeup of the virus through mutation and genomic reassortments are important factors in the emergence of tospoviruses.

# Research article: Using epidemiological information to help manage tospoviruses effectively: the experience with *Tomato spotted wilt virus* in Australia

**Published:** 2010-10-01

**Authors:** Roger A.C. Jones, Brenda Coutts

**Affiliations:** Plant Pathology Section, Agricultural Research Western Australia, Department of Agriculture and Food, Bentley Delivery Centre, WA 6983, and School of Plant Biology, M084, Faculty of Natural and Agricultural Sciences, University of Western Australia, Crawley, WA 6009, Australia

The diseases caused by thrips-transmitted tospoviruses (genus *Tospovirus*, family *Bunyaviridae*) are a major constraint to production of important vegetable, legume and ornamental crops in different parts of the world. Their often wide and overlapping host ranges, emergence of resistance-breaking strains, circulative and propagative relationship with polyphagous thrips vectors, and difficulties in predicting their outbreaks pose challenges to development and implementation of effective management programmes. Despite these challenges, for a few tospoviruses, considerable progress has been made in successful development and deployment of practical and effective integrated disease management (IDM) programmes. This has been due to increased understanding of their epidemiology, identification of risk factors that contribute to increased disease incidence and development of tactics to mitigate those risk factors. This paper will focus as an example on how epidemiological information on *Tomato spotted wilt virus* and field experiments on potential control measures helped in devising an effective Integrated Disease Management Strategy for this virus in Australia.

# Research article: Extension strategies for improved Western flower thrips management in Queensland

**Published:** 2010-10-01

**Authors:** Iain Kay, S. Heisswolf

**Affiliations:** ^1^ Queensland Primary Industries and Fisheries, 49 Ashfield Road, Bundaberg, Qld. 4670, Australia; ^2^ Queensland Primary Industries and Fisheries, PO Box 538, Bowen, Qld. 4805. Australia

*Frankliniella occidentalis* (Pergande), western flower thrips (WFT), has been an important pest of vegetable crops in Queensland since 2001, causing damage through its feeding and by vectoring *Tomato spotted wilt virus* (TSWV). In 2003 Horticulture Australia commissioned a project to transfer WFT/TSWV management technology developed during a decade of research in southern Australia to vegetable growers in the Bundaberg and Bowen-Burdekin districts of Queensland. Project research activities focussed on adapting information and technology derived from work in southern states to north Australian conditions. This included defining the seasonal occurrence of WFT, identifying important alternative hosts, and testing insecticides for efficacy against WFT. The work was supported by related studies by other researchers on monitoring insecticide resistance in WFT and on better managing TSWV and other tospoviruses. A survey identified the issues and needs of the industry in the target districts and shaped the extension approach used. Four interrelated approaches were used. Presentations and discussions at workshops, seminars and field days on topics such as thrips identification and biology, weed hosts and crop hygiene, insecticide use and application were often combined with sessions on managing other pests. A newsletter, WFT Queensland Update, was produced and distributed to growers and agribusiness service providers, as was other printed information. Project team members worked closely with growers who often prefer one-on-one contact to access information on pest and disease management. Collaboration with established commercial consulting firms in the project enabled fast integration of thrips and disease information in the integrated pest management strategies they promote, and ensured the longterm provision of WFT/TSWV management advice in each district. An independent, external end of project evaluation determined that there was a high level of awareness of the project, its key messages and the practices it promoted among growers and service providers.

# Research article: The aggregation pheromones of thrips

**Published:** 2010-10-01

**Authors:** William D.J. Kirk, James G. C. Hamilton

**Affiliations:** School of Fife Sciences, Keele University, Staffordshire ST5 5BG, United Kingdom

Adult males of many species of thrips (Thysanoptera) have externally visible pore plates (*areae porosae*) on their abdominal sternites. In the sub-family Thripinae, the plates are typically on sternites III–VII. A few of these species have been studied microscopically and they have large, internal, glandular structures associated with the pore plates, indicating that the function of the plates is to release pheromone. Identification and characterisation of male-produced pheromones is difficult because they are not stored, but produced on demand in very small (picogram) amounts. In the western flower thrips *Frankliniella occidentalis* (Pergande), there are two major compounds that are produced only by males. One of these, neryl (*S*)-2-methylbutanoate, increases trap catches of males and females in glasshouses and so acts as an aggregation pheromone. A synthetic form of the pheromone is sold commercially by Syngenta Bioline under the name Thripline_ams_ to increase trap catches of *F. occidentalis* in protected crops. Growers have also observed that thrips become more active on the surface of the crop when the pheromone is released. The role of the second compound, (*R*)-lavandulyl acetate, remains unclear. We have identified, but not fully characterised, a male-produced compound in the melon thrips *Thrips palmi* Karny. It appears to be a monoterpenoid pentenoate (RM=236) and is very similar in structure to the aggregation pheromone of *F. occidentalis*, which is a monoterpene pentanoate (RM=238). We predict that this is also an aggregation pheromone. The similarity is remarkable given that the genera *Frankliniella* and *Thrips* probably diverged between about 120 million and 70 million years ago. The presence of a second male-produced compound in *F. occidentalis*, but not in *T. palmi*, may relate to differences in mating behaviour. For example, aggressive interactions between males are known in *F. occidentalis* and *Frankliniella intonsa* (Trybom), but not so far in any *Thrips* species.

# Research article: Plant compounds for modifying the behaviour of *Thrips tabaci*

**Published:** 2010-10-01

**Authors:** Elisabeth H. Koschier, J. Riefler

**Affiliations:** Institute of Plant Protection, Department of Applied Plant Sciences and Plant Biotechnology, University of Natural Resources and Applied Fife Sciences (BOKU) Vienna, Peter Jordan-Straße 82, 1190 Vienna, Austria

*Thrips tabaci* Lindeman (Thysanoptera: Thripidae) is a cosmopolitan pest species causing serious losses in many protected crops as well as in the field. Previous studies have identified the secondary plant compounds linalool, a monoterpene, and eugenol, a phenylpropanoid, as feeding and oviposition deterrents against the onion thrips. Because these results were obtained exclusively in end point bioassays, changes in behavioural patterns of *T. tabaci* in response to the deterrents remained unknown. In the present study behavioural observations of thrips that did not take flight over a complete photoperiod revealed that a foliar application of linalool or eugenol to leek not only modified the feeding and oviposition behaviour of *T. tabaci* adult females, but also influenced their movement patterns and prolonged the periods of inactivity of thrips on treated plant surfaces. The increased understanding of the mechanisms of linalool and eugenol as signal chemicals make them valuable model compounds in a future search for effective compounds as synergists, for example in the framework of a push-pull strategy and various other biological or chemical control measures in conventional, integrated or organic farming systems.

# Research article: The behavioural repertoire of *Thrips tabaci*

**Published:** 2010-10-01

**Authors:** Elisabeth H. Koschier, J. Riefler

**Affiliations:** Institute of Plant Protection, Department of Applied Plant Sciences and Plant Biotechnology, University of Natural Resources and Applied Fife Sciences (BOKU) Vienna, Peter Jordan-Straße 82, 1190 Vienna, Austria

This study describes all behavioural patterns and activities of *Thrips tabaci* Lindeman (Thysanoptera: Thripidae), the onion thrips. This thrips species is a cosmopolitan pest that causes losses in many protected crops as well as in the field. *Thrips tabaci* females were observed on small rectangles cut from host plant leaves that were placed on water agar cubes as a water supply in water-filled Petri dishes fixed under a microscope. This setup confined single thrips females to the observational arenas while allowing them to move freely on a piece of plant tissue, with the surrounding water keeping them from leaving. Thrips behaviours were recorded continuously for up to 24 hours (L:D = 16:8) using a microscope with a cold light source or an infra-red illumination, a video camera and a video recorder.

Considering descriptions of specific behaviours of thrips in the literature, 17 behavioural elements shown by adult females were videotaped, described and classified into 7 behavioural categories: inactivity, exploratory behaviour, oviposition behaviour, feeding behaviour, defecation, cleaning behaviour and take-off behaviour.

Using this classification, differences in host use on different host plants of the onion thrips and/or behavioural responses of thrips to bioactive plant compounds can be assessed.

# Research article: Biological and molecular characterisation of vegetable infecting tospoviruses in India

**Published:** 2010-10-01

**Authors:** S.R. Kunkalikar, P Sudarsana, M. Bhanupriya, Prem A. Rajagopalan, S.D. Yeh, R.A. Naidu, Usha B. Zher, K.S. Ravi

**Affiliations:** ^1^ Molecular Virology, Mahyco Research Center, Dawalwadi, Post Box no. 76, Jalna, Maharashtra - 431203, India; ^2^ Department of Plant Pathology, National Chung Hsing University, Taichung, Taiwan, R.O.C.; ^3^ Department of Plant Pathology, Irrigated Agriculture Research and Extension Center, Washington State University, Presser, WA 99350. USA

Three tospovirus species (*Peanut bud necrosis virus* (PBNV); *Watermelon bud necrosis virus* (WBNV) and *Peanut yellow spot virus* (PYSV) have been reported from India since 1992. However, a detailed analysis of the biological and molecular properties of these viruses has not yet been carried out. Therefore, as a first step towards the characterisation of these viruses and the development of management strategies based on host resistance, we carried out a country-wide survey for the distribution and genetic diversity of vegetable-infecting tospoviruses. Disease incidence and virus infected plant samples with typical tospovirus symptoms have been collected from 12 different states, comprising the major vegetable growing areas of the country since 2002. Based on serological cross relationship, sequence comparisons and phylogenetic analyses, at least two previously unreported species (*Iris yellow spot virus* (IYSV) and *Capsicum chlorosisc virus* (CaCV)), were discovered from India by our group. Natural and biological host ranges of these viruses were compared. N gene of 85 isolates of PBNV, 38 isolates of WBNV, five isolates of CaCV and 26 isolates of IYSV were characterised. Further the mixed infection of *Watermelon silver mottle virus* (WSMoV) serogroup viruses and/or with other viruses (*Cucumber mosaic virus* (CMV), *Tobacco mosaic virus* (TMV), and *Chilli venal mottle virus* (ChVMV) in hot pepper and *Tomato leaf curl virus* (ToLCV) in tomato were studied. Interestingly we have noticed high level synergisms with other group of viruses and cross protection among the WSMoV serogroup viruses. As a result of surveys conducted from 2002 to 2009 in major tomato growing areas of Hoskote district of Karnataka states (hot spot to PBNV), a total of 23 N genes were characterised at a regular intervals. The high conservation in the N gene sequences were noticed when compared with a PBNV isolate, which had been first characterised nearly a decade ago indicates that the PBNV N gene continues to be conserved over a period of nearly 15 years in endemic regions. Further the full length of S and M genomic segments of WBNV and CaCV were characterised.

# Research article: Thrips profiles on Scotch bonnet pepper (*Capsicum Chinese*) and tomato (*Lycopersicon esculentum*) in Uganda

**Published:** 2010-10-01

**Authors:** Samuel Kyamanywa, M.K.N.S. Ochwo, J. Karungi, Naidu A. Rayapati, C. Ssemwogere

**Affiliations:** ^1^ Department of Crop Science, Makerere University, P. O. Box 7062, Kampala; ^2^ Department of Plant Pathology, Washington State University P. O. Box 646430, USA

Tomatoes (*Lycopersicon esculentum*) and Scotch bonnet pepper are important sources of cash for small scale farmers in Uganda. The two crops, however, are devasted by several viral diseases which are transmitted by thrips. The epidemiology of thrips is not well understood. Consequently there are no recommended control practices for the viruses and their vectors on the two crops. A study was therefore initiated to determine the incidence of different thrips species on tomatoes and pepper and how they are influenced by production practices. The study was conducted in farmers' fields in Mpigi and Wakiso districts. Initial identification indicated that there were five different species of thrips in the farmers' fields which included: *Frankliniella occidentalis, Frankliniella schultezei, Thrips tabaci, Scirtothrips dorsalis* and *Thrips palmi*. Three of the species (*F. Occidentalis, F. Shultezei* and *T. tabaci*) were found only on tomatoes while the other two species (*T. palmi* and *F. occidentalis*) were found on both. The five thrips were significantly affected by cropping systems and location. This paper uses current ecological principles to discuss the implication of the present results on thrips management and their impact on production of the two crops in Uganda.

# Research article: Evaluation of *Tomato spotted wilt virus* resistant bell pepper lines in Georgia, USA

**Published:** 2010-10-01

**Authors:** David Langston, David Riley

**Affiliations:** University of Georgia, Athens, GA 30602, USA

Thrips-vectored *Tomato spotted wilt virus* (TSWV) is one of the more severe problems affecting pepper production in the Southeastern USA. Currently, commercially available pepper cultivars resistant to TSWV are mostly based on a single gene; *tsw* gene in pepper. These resistant lines are available and to some extent are grown commercially in the southeastern USA. However, many of these TSWV-resistant cultivars have horticultural characteristics that make them less desirable than susceptible traditional hybrids. Also, resistance-breaking strains of TSWV have occurred in many areas where resistant cultivars have been grown (Moyer and Kennedy, unpublished, Thomas-Carroll and Jones 2003, Aramburu 2003), emphasizing the need for regional evaluations of TSWV resistant pepper cultivars. In pepper evaluations conducted during the spring season at Tifton, Georgia from 2006–2008, the pepper cultivar ‘Heritage’ from Harris Moran Seed Company was consistently the highest yielding bell pepper and averaged less than 3% TSWV symptoms over the three years.

The pepper lines ‘Plato’ from Seminis and ‘Magico’ from Harris Moran both averaged less than 1% symptomatic and were also high yielding. ‘Stiletto’ from Syngenta also averaged 1% symptomatic, but yields were significantly lower than the previously mentioned resistant lines.

# Research article: *Tomato spotted wilt virus* NSm protein domains involved in tubule formation, movement and symptoms

**Published:** 2010-10-01

**Authors:** Weimin Li, Dennis J. Fewandowski, Mark E. Hilf, Scott Adkins

**Affiliations:** ^1^ Institute of Plant Virology, Fujian Agricultural and Forestry University, Fuzhou 350002, PRC; ^2^ Department of Plant Pathology, Ohio State University, Columbus, OH 43210, USA; ^3^ USDA-ARS, Fort Pierce, FF 34945, USA

Direct demonstration of *Tomato spotted wilt virus* (TSWV) gene function has been slowed by the absence of a reliable reverse genetics system. A *Tobacco mosaic virus* (TMV)-based expression system was previously used by us to demonstrate that the TSWV NSm protein is able to support cell-to-cell movement in the absence of any other TSWV proteins. Further demonstration that NSm induced tubule formation in protoplasts, supported long-distance movement and induced TSWV-like symptoms in plants was also facilitated by the TMV-based expression system. Essential NSm domains for tubule formation, movement and symptoms have now been identified by our use of deletion-mapping and alanine-substitution mutagenesis via the TMV-based system. Two regions of NSm were required for both tubule formation in protoplasts and cell-to-cell movement in plants, indicating a correlation between these activities. Results of our mutagenesis studies of conserved amino acids suggest that the function(s) predicted from domains common to tospovirus NSm proteins may be conserved across the genus. Further exploration of functions of this interesting protein from both additional TSWV isolates and other tospovirus species is merited.

# Research article: Detecting a *Tospovirus* in trapped thrips

**Published:** 2010-10-01

**Authors:** R. Lister, M-C. Nielsen, Melanie Davidson, J. Fletcher, G. Timmerman-Vaughan

**Affiliations:** Plant and Food Research, Private Bag 4704, Christchurch, New Zealand

Only three of the 14 *Tospovirus* species and three of their 11 known thrips vector species have been recorded in New Zealand. In New Zealand *Frankliniella occidentalis*, and *Thrips tabaci* vector *Tomato spotted wilt virus* (TSWV), while *T. tabaci* vectors *Iris yellow spot virus* (IYSV) and *Impatiens necrotic spot virus* (INSV) is vectored primarily by *F. occidentalis. Frankliniella occidentalis* is also known to vector three other tospoviruses not yet recorded in New Zealand. The importance of other thrips species as virus vectors in New Zealand is not yet clear. Thus we not only have to monitor our borders for new potentially pestiferous thrips species that may be capable of vectoring tospoviruses but also for the tospoviruses themselves. We found that by using ELISA and RT-PCR techniques it was possible to detect the presence of TSWV from thrips (*F. occidentalis*) that had been trapped on sticky boards for 24 h, and after 1, 2, 3, or 4 weeks. The implications of this technique to detect a new virus or vector incursion, are discussed.

# Research article: Sequencing of New Zealand's *Tomato spotted wilt virus* isolates

**Published:** 2010-10-01

**Authors:** R. Lister, G. Fimmerman-Vaughan, John Fletcher

**Affiliations:** Plant and Food Research, Private Bag 4704, Christchurch, New Zealand

The capsid protein genes of thirteen isolates of *Tomato spotted wilt virus* (TSWV) (*Tospovirus*) occurring in New Zealand have been sequenced. With recent new incursions of thrips species and tospoviruses it was considered of interest to identify any sequence variation occurring in the New Zealand TSWV isolates, both from a temporal and geographic perspective. The material chosen for sequencing was varied. The oldest TSWV isolate was collected in 1992 and stored frozen since then, and the most recently collected was from fresh material in 2008. Samples were obtained from several geographic locations throughout New Zealand. When compared to the world-wide TSWV capsid gene sequence data held in GenBank, cluster analysis of the sequence data has placed the New Zealand isolates into distinct groups. Observations of the most closely related strains on a global scale may provide clues to the country of origin of historic TSWV incursions into New Zealand.

# Research article: Western flower thrips in China

**Published:** 2010-10-01

**Authors:** Yaobin Lu, Zhijun Zhang

**Affiliations:** Institute of Plant Protection and Microbiology, Zhejiang Academy of Agricultural Sciences, 198 Shiqiao Road, Hangzhou, Zhejiang 310021, China

Western flower thrips (WFT), *Frankliniella occidentalis*, is a serious pest in many countries and regions around world. This pest was first recorded on the China mainland in 2003. Since that time this pest has been found in several provinces and has caused serious damage to vegetable and ornamental crops in some regions in China. In order to effectively control this invasive alien pest and prevent its spreading nationwide, the Chinese government has made great efforts. In order to further improve the research on this pest, the Chinese government funded a large project for the sustainable control of this pest in 2008. The budget is 2007 CNY (about 3 million USD). As the leader of this national key project I have established a workshop to fight against this serious pest. More than 60 experts from 12 different institutions around China participate in this project. In order to seek more help and experience from other countries to solve the problems caused by WFT in China, and to develop collaboration opportunities, I will introduce this project to other conference participants so that they will know about what we have done and what we will do on this pest.

# Research article: *Tomato spotted wilt virus* strains expressing a truncated NSs protein can not be transmitted by the thrips vector *Frankliniella occidentalis*

**Published:** 2010-10-01

**Authors:** P. Margaria, M. Ciuffo, F. Bosco, G. Mautino, F. Tavella, Massimo Turina

**Affiliations:** ^1^ Istituto di Virologia Vegetale, Sez. di Torino, CNR, Strada delle Cacce n73, Torino 10135, Italy; ^2^ Di.Va.P.R.A. Entomologia e Zoologia applicate all'Ambiente, University of Torino, Via L. da Vinci 44, 10095 Grugliasco (TO), Italy

In standard leaf disk transmission assays, *Frankliniella occidentalis* adults were unable to transmit *Tomato spotted wilt virus* (TSWV) strains p170RB and p202 coding for a truncated NSs protein and described in a previous work (Margaria et al., 2007). In order to characterise NSs-defective non-transmissible strains, we forced the obtainment from a wild-type strain (p202/3WT) of a NSs truncated strain by a single mechanical inoculation on *Capsicum chinensis* ‘PI152225’. Selective pressure of the *Tsw* resistance gene allowed to obtain isolates carrying mutations in the NSs coding region: among them, we looked for strains undergoing symptom recovery, which we previously showed to be associated to deletions in the NSs protein. The strain we obtained was named p202/3RB and carried a single nucleotide mutation (G deletion) in the NSs coding region, resulting in a frame-shift and a truncated NSs protein of 443aa (compared to 467aa of p202/3WT). When transiently expressed in *Nicotiana benthamiana* leaves, the truncated protein accumulated, but could not suppress silencing. The two strains were tested for their transmissibility by *F. occidentalis*, obtaining 53.3% of transmission for p202/3WT and 0% for p202/3RB. Quantitative RT-PCR assays on the adult thrips immediately after the transmission assays allowed us to detect a very low titre of viral RNA for p202/3RB compared to a much more abundant p202/3WT viral RNA. Sequence comparison of the genomic segments of the two related strains showed 100% homology for the S (small) segment with the exception of the G deletion and 100% homology for the M (medium) segment. Moreover, we obtained a reassortant strain, carrying the L (large) segment from a transmissible isolate (p105) and M-S segments from p202/3RB, which was not transmissible, allowing us to exclude that the L segment is involved in transmission efficiency. Taken together our data provide genetic evidence for the involvement of the S segment in the TSWV-thrips relationship and for a role of NSs in efficient vector infection.

# Research article: Managing western flower thrips (*Frankliniella occidentalis*) and *Tomato spotted wilt virus* in lettuce in Australia

**Published:** 2010-10-01

**Authors:** Sandra McDougall

**Affiliations:** NSW DPI, Vegetable Industry Centre, Yanco NSW 2703, Australia

Until the arrival of Western flower thrips (WFT: *Frankliniella occidentalis*) in Australia in the early 1990s *Tomato spotted wilt virus* (TSWV: *Bunyaviridae Tospovirus*) had been a sporadic pest of lettuce (*Lactuca sativa* L.), vectored by Tomato thrips (*Frankliniella schultzei*) and Onion thrips (*Thrips tabaci*). The arrival of WFT into lettuce production areas, particularly those adjacent to capsicum (*Capsicum annuum*) or tomato (*Lycopersicon esculentum*) saw an increase incidence and severity of TSWV. Despite the limited chemical options growers in these areas have been some of the slowest to adopt the most basic of Integrated Pest Management (IPM) tools but perhaps have the most to gain. This talk will give a national context on how WFT and TSWV have impacted on the development and adoption of an IPM strategy in lettuce. A broad overview will be presented on R&D responses, identify the challengers and present potential solutions.

# Research article: Screening for *Tomato spotted wilt virus* resistance in potatoes

**Published:** 2010-10-01

**Authors:** M. Milinkovic, T. Slater, Brendon Rodoni

**Affiliations:** Department of Primary Industries, Knoxfield Centre, 621 Burwood Highway, Knoxfield, Vic, 3156, Australia.

*Tomato spotted wilt virus* (TSWV) causes significant yield losses in seed and commercial potato crops across Australia. The Australian environment provides an abundance of thrips vectors and alternative weed hosts for both TSWV and the vectors, and therefore the control of TSWV is difficult in many districts. Chemical control is ineffective, expensive and is often not compatible with IPM programs. This problem is unique to Australia and as a result there has been very little work towards the development of TSWV resistance in potato germplasm by breeding programs around the world. TSWV resistant potato cultivars could provide an effective control strategy for this disease by protecting the plant against viriliferous thrips flying onto the crop from surrounding weeds. A TSWV-resistance screening protocol and disease resistance scale has been developed to be used routinely to screen potato germplasm for TSWV resistance in the Australian potato breeding program. Forty (40) potato breeding parents have been challenged and scored and we have identified 9 TSWV resistant and 9 TSWV susceptible cultivars. Families have been screened that have resistant and susceptible parents to identify segregating phenotypes. A suitable family will be developed to identify a molecular marker for TSWV resistance. Ultimately this molecular marker will be used to rapidly screen breeding progeny for the development of resistant cultivars. Observations from repeated challenges of TSWV on a large number of potato cultivars have identified considerable variation between cultivars in the translocation of TSWV within a potato plant and further studies are required to understand this phenomenon.

# Research article: Biological diversity of Iranian Haplothripini (Thysanopter: Phlaeothripidae)

**Published:** 2010-10-01

**Authors:** Kambiz Minaei

**Affiliations:** Plant Protection Department, College of Agriculture, Shiraz University, Shiraz, Iran

The 5800 described species of Thysanoptera exhibit a wide range of biologies. About 50% feed on fungi, with most of these feeding on hyphae but some on spores. Of the remainder, approximately equal numbers feed either in flowers or on green leaves. About 100 species are crop pests, causing feeding damage and vectoring tospoviruses, but sometimes acting as beneficials by feeding on other pest arthropods or pollination.

In Iran, four genera and 27 species are recorded of the Thysanoptera tribe Haplothripini (Phlaeothripidae, Phlaeothripinae). *Dolicholepta* Priesner with one species, *Haplothrips* Amyot and Serville with 23 species, *Neoheegeria* Schmutz with two species and *Plicothrips* Bhatti with one species. *Haplothrips* species have a diversity of biologies, with many species apparently predatory and others phytophagous, particularly in the flowers of Asteraceae and Poaceae and a few are pest. In contrast, most other Haplothripini found in this country seem to be phytophagous and host specific on other plant families. Larvae and adults of *Dolicholepta micrura* (Bagnall) have been found in Iran only on the leaves of *Zizyphus spina-christi*. The available biological data about *Neoheegeria dalmatica* Schmutz and *N. persica* Priesner suggest that these species are associated with the flowers of Lamiaceae, although this host association is possibly not strict. *Plicothrips apicalis* (Bagnall) occurs in the inflorescences of *Cynodon dactylon* (Poaceae).

In general, Haplothripini species of Iran are commonly associated with the flowers of Asteraceae and Poaceae, but with some species having particular associations with other plants.

# Research article: Structural variation and identification problems in the wheat thrips, *Haplothrips tritici* (Thysanoptera: Phlaeothripidae)

**Published:** 2010-10-01

**Authors:** Kambiz Minaei, Laurence Mound

**Affiliations:** ^1^ Plant Protection Department, College of Agriculture, Shiraz University, Shiraz, Iran; ^2^ CSIRO Entomology, Canberra, Australia

Wheat thrips, *Haplothrips tritici* (Kurdjumov) is a common pest of cereals in various parts of Europe and Iran. The morphological recognition of this species is by no means secure, in particular the distinction from *Haplothrips cerealis* Priesner that is commonly recorded from cereal crops in several countries. The objectives of the present work were to reconsider the significance of several morphological character states that are commonly used in taxonomic work on *Haplothrips* species, to record the variation in a number of characters within several populations of ‘wheat thrips’ in Iran, and to determine whether this variation seems likely to represent more than one species. Six populations of *Haplothrips* wheat thrips collected from three provinces of Iran were analysed morphologically, using both numerical and descriptive character states, and the considerable variation within and between populations is recorded. Differences frequently quoted to distinguish *H. cerealis* from *H. tritici* are shown to occur within these six populations of wheat thrips from Iran. This suggests that these populations represent variants of a single species. Therefore, *H. tritici* is the only species of *Haplothrips* that is common on Poaceae in Iran, and records of *H. cerealis* from Iran are probably misidentifications. The extensive variation within these populations in numerical and descriptive characters has broad implications for species taxonomy, particularly within *Haplothrips*, the third largest genus of Thysanoptera.

# Research article: Sources of onion thrips infestations in onions in NSW and SA

**Published:** 2010-10-01

**Authors:** Jianhua Mo, G. Baker, G. Herron, L. Mound

**Affiliations:** ^1^ Yanco Agricultural Institute, NSW Department of Primary Industries, Yanco, NSW 2703, Australia; ^2^ Entomology Unit, South Australian Research and Development Institute, GPO Box 397, Adelaide, SA 2570, Australia; ^3^ Elizabeth MacArthur Agricultural Institute, NSW Department of Primary Industries, PMB 8, Camden 2570, Australia; ^4^ CSIRO Div. of Entomology, Clunies Ross Street, Acton, Canberra, NSW 2601. Australia

Non-onion hosts of onion thrips (*Thrips tabaci*) in the major onion (*Allium sepa*) production regions of NSW and SA were surveyed during March – October in 2005 and 2006. A total of 40 plant species from 17 families were surveyed. Onion thrips adults were found in 32 species from 15 families and larvae in 24 species from 12 families. The families Brassicaceae and Asteraceae accounted for most of the plants where onion thrips were found. From March to onion emergence in mid June–July, onion thrips were mostly abundant on Indian hedge mustard (*Sisymbrium orientale*), blackberry nightshade (*Solanum nigrum*), and twiggy turnip (*Brassica fruticulosa*). During the early part of onion seasons, onion thrips were most abundant on shepherd's purse (*Capsella bursapastoris*), Indian hedge mustard, and capeweed (*Arctotheca calendula*) in NSW, and on horehound (*Marrubium vulgare*), variable groundsel (*Senecio lautus*), and onion weed (*Asphodelus fistulosus*) in SA. Among crops widely planted in the survey areas, onion thrips larvae were found in Faba Bean (*Vicia faba*) and canola (*Brassica napus var. napus*) but not in wheat (*Triticum aestivum*) and soybean (*Glycine max*), both being confirmed hosts. In contrast to the sporadic presence of weed hosts, wheat, Faba Bean and canola are planted in large areas alongside onions and are harvested well before onions, making them potentially significant sources of onion thrips infestations in onions.

# Research article: A review of the epidemiological history of TSWV in Georgia, USA: where we are now

**Published:** 2010-10-01

**Authors:** Stephen W. Mullis, A.S. Csinos, C. Nischwitz, R.D. Gitaitis

**Affiliations:** ^1^ Department of Plant Pathology, University of Georgia, Tifton, GA, 31793-0748, USA; ^2^ Division of Plant Pathology and Microbiology, University of Arizona, Fucson, AZ, 85721-0036, USA

Although first reported in Georgia in 1986, *Tomato spotted wilt virus* (TSWV) was not considered a problem until 1989. Since that time, farm gate losses in agricultural community have been devastating. It has been common to have losses approaching 90%. The southern Georgia farmscape offers an environment highly conducive to disease and insect pressure. The cultivation of several susceptible crops in close proximity to each other, the year-round availability of numerous, non-crop, host species, the widespread presence of thrips vectors such as *Frankliniella occidentalis* and *F. fusca*, and the temperate environment conducive to TSWV infection cycles makes the control of the disease difficult. Elimination of the thrips vectors or inoculum sources are not viable options in the management of TSWV, so efforts have been focused on integrated management programs for the mitigation of the TSWV losses. Risk indices have been incorporated in to peanut crop management systems. Variables that lower TSWV risk including varied cultivation techniques, planting dates and use of resistant crop varieties are practices have been effective at reducing the impact of TSWV in pepper and tomato. Tobacco has not been as responsive to these cultural practices, and efforts to deploy control options such as insecticide sprays, adjusting planting dates, rouging of infected plants, and replanting have had limited and unpredictable results on reducing TSWV infections. However, there has been some success in tobacco disease management with the employment of the compound Actigard, which induces systemic acquired resistance. There is still much that is not known about the epidemiology of TSWV. The roles that the environment, farmscape, and thrips populations, as well as thrips-plant interactions and intra-plant disease physiology are variables that all play a role in the TSWV cycle, and the relations that all of these variables offer to the TSWV disease dynamic are still poorly understood.

# Research article: Damage to vegetable crops by *Thrips parvispinus* Karny (Thysanoptera: Thripidae) and preliminary studies on biology and control

**Published:** 2010-10-01

**Authors:** Famotsu Murai, Hiroki Watanabe, Wataru Foriumi, Faro Adati, Shuji Okajima

**Affiliations:** ^1^ Utsunomiya University, 350 Mine, Utsunomiya, Japan 321-8505; ^2^ Tokyo University of Agriculture, 1-1-1, Sakuragaoka, Setagaya-ku, Tokyo Japan 156-8502

*Thrips parvispinus* Karny is widely distributed in the Pacific region; however, little is known of its biology. No damage by this thrips was described. We found damage by this thrips occurred in several vegetable crops, such as chili pepper, paprika, green bean, potato, strawberry, eggplant in Bali and Bogor in Indonesia. Especially, there was heavy damage on chili pepper and strawberry. Similar damage by this thrips was observed in chili pepper. However, no damage was observed on cucumber and eggplant in Rachabru in Thailand. *T. parvispinus* became the dominant thrips species in Indonesia, replacing *Thrips palmi* in several vegetable fields. We preliminarily tested several colour sticky traps as a monitoring method for this thrips. *T.parvispinus* was likely to be attracted to white rather than blue or yellow. Development and reproduction of *T. parvispinus* were tested at 20°, 25° and 30°C condition. Developmental zero was 12.7 and thermal constant was 144.9 day degrees. Mean fecundity and mean generation time at 20°, 25° and 30°C were 50, 69, and 56 eggs and 37.6, 24.8 and 18.8 days, respectively. Intrinsic rate of natural increase at 20°, 25° and 30°C was 0.18, 0.24 and 0.37, respectively. Some chemicals were tested to evaluate conventional potential by leaf dipping method. *T.parvispinus* was susceptible to spinosad but had low effect to acetamiprid. Status of *T. parvispinus* as pest thrips is likely to be replaced by *T. palmi*. We are afraid of dispersion and developing resistance to chemicals of this thrips species. At the moment we are testing virus transmission such as TSWV and CaCV.

# Research article: Effects of temperature and host plant on wing-form determination of a Japanese strain of the tobacco thrips, *Frankliniella fusca* (Hinds) (Thysanoptera: Thripidae)

**Published:** 2010-10-01

**Authors:** Shiro Nakao, C. Chikamori

**Affiliations:** Kyoto Prefectural University, Shimogamo 1–5, Sakyo-ku, Kyoto 606-8522, Japan

Recently, the tobacco thrips, *Frankliniella fusca* (Hinds) were twice found in bulbs of the narcissus at garden centres in Japan. To clarify the effects of temperature and food type on wing-form determination in one of the two strains, the thrips were reared from hatching to adult eclosion on leaf pieces of green bean, broad bean and narcissus and on narcissus bulbs and germinated broad bean seeds under long-day photoperiodic conditions (15L-9D) at 18°C, 22.5°C, and 25°C. All adult males were brachypterous in the present study, and all females developing at 18°C emerged as brachypters irrespective of food types. The percentages of macropters in females developing at 25°C were apparently higher than those at 22.5°C. Under 25°C, percentage of macropters in females was the highest on germinated broad bean seeds (ca. 81%), and lowest on narcissus bulbs (ca. 8%). On leaf pieces of the three plant species, 14–32% of females developed into macropters at 25°C. Thus, temperature and food type obviously affected wing-form of females in the Japanese strain of the tobacco thrips. In addition to these findings, it was suggested that photoperiodism does not have an effect on the wing-form determination, and females of this strain did not enter reproductive diapause even under short-day photoperiod (10L-14D and 18∼25°C).

# Research article: Life history and fighting behaviour of an Australian gall-inducing thrips, *Kladothrips rodwayi* Hardy (Thysanoptera: Phlaeothripidae), with a discussion about the loss of solders in *Acacia thrips*

**Published:** 2010-10-01

**Authors:** Shiro Nakao, M. Schwarz

**Affiliations:** ^1^ Kyoto Prefectural University, Shimogamo 1-5, Sakyo-ku, Kyoto 606-8522, Japan; ^2^ Flinders University of South Australia, GPO Box 2100, Adelaide, SA 5001, Australia

To detect possible reasons why one of the Australian *Acacia thrips, Kladothrips rodwayi* Hardy (Thysanoptera: Phlaeothripidae) has lost the solder generation, (1) inter-population variations of annual life-cycle of *K. rodwayi*, and (2) inter-population variations of kleptoparasite pressure by *Koptothrips flavicornis* Bagnall (Thysanoptera: Phlaeothripidae) were investigated in SA, VIC and TAS from December 2006 to August 2007, and furthermore, (3) proclivity and effectiveness in gall defence by foundresses (dispersers) were experimentally assessed in laboratory.

In general, percentages of successful kleptoparasitism were higher in *K. rodwayi* galls at low latitudes and lower altitude site (e.g. ca. 60% at two sites in SA and Heywood in VIC, 20% or below at Halls Gap and Portland in VIC and all sites in TAS). Foundresses of *K. rodwayi* showed obvious proclivity to attack kleptoparasites, but the fighting behaviour of foundresses does not seem to effective for gall defence against *Koptothrips* invaders. On the other hand lethal fighting between foundresses of *K. rodwayi* was suggested by observations on broods in galls from TAS. Based on these results and annual life-cycles of *K. rodwayi* and *Ko. flavicornis* in different areas, we reviewed previous studies (Crespi and Abbot, 1999: Kranz et al., 2002). The loss of solders in *K. rodwayi* might be associated with adaptability to mesic and cooler conditions with decreasing accumulated temperature during their development and periods for kleptoparatitism.

# Research article: Controlling onion thrips in onion with insecticides

**Published:** 2010-10-01

**Authors:** Brian Nault, C. Hsu, A. Shelton

**Affiliations:** Cornell University, Department of Entomology, New York State Agricultural Experiment Station, 630 W. North St., Geneva, NY 14456, USA

Insecticides are the primary tool for managing onion thrips, *Thrips tabaci* Lindeman (Thysanoptera: Thripidae), in onion fields. Some insecticides used for thrips control perform adequately, while others perform poorly. Several reasons that may explain poor performance include a weak active ingredient, a short residual activity, an application made too late, or a resistant thrips population. Any one of these reasons can result in explosive populations and significant economic losses. To address some of these issues, our research focused on two objectives: (1) identify new effective insecticides that control onion thrips, and (2) determine action thresholds for timing insecticides to manage thrips. From 2005–2009, 22 active ingredients were evaluated for onion thrips control in field trials. Only five of these products were considered highly effective against onion thrips and two are now registered in the US. From 2006–2008, action thresholds of 1, 3 and 5 larvae/leaf were evaluated for four products: lambda-cyhalothrin, methomyl, formetanate hydrochloride and spinetoram. The best action threshold, based on the level of thrips control, feeding damage and marketable yield, was dependent on the product. For methomyl, a threshold of 1 larva/leaf provided acceptable control. For formetanate hydrochloride and spinetoram, a threshold of 3 larvae/leaf was acceptable. Lambda-cyhalothin did not control thrips regardless of the threshold used. Waiting to apply any insecticide until thrips populations reached 5 larvae/leaf resulted in poor control, unacceptable levels of damage, and sometimes low yields. These results combined with insecticide resistance management principles are being used to develop a season-long thrips management program.

# Research article: Ecology of onion thrips and epidemiology of *Iris yellow spot virus*: Implications for management in New York onion fields

**Published:** 2010-10-01

**Authors:** Brian Nault, C. Hsu, A. Shelton, M. Fuchs, C. Hoepting

**Affiliations:** ^1^ Cornell University, Department of Entomology, New York State Agricultural Experiment Station, 630 W. North St., Geneva, NY 14456; ^2^ Cornell University, Department of Plant Pathology Plant Microbe Biology, New York State Agricultural Experiment Station, 630 W. North St., Geneva, NY 14456; ^3^ Cornell Cooperative Extension, Cornell Vegetable Program, 12690 Rte. 31, Albion, NY 14411, USA

Onion thrips, *Thrips tabaci* Lindeman (Thysanoptera: Thripidae), is the major insect pest of onion in New York. Thrips can reduce bulb yields by at least 33% and they transmit *Iris yellow spot virus* (IYSV), which can cause additional yield losses. In New York, onions are planted in April using virus-free seeds or transplants, but there are a number of alternative plant hosts for IYSV and thrips that serve as a green bridge for IYSV in the spring. Transplants are larger than direct-seeded onions early in the season and thrips may preferentially colonise larger plants, resulting in earlier infections and higher levels of IYSV in transplanted fields. Alternatively, most transplanted fields are harvested before direct-seeded fields, possibly before IYSV reaches high levels. Viruliferous thrips may migrate from these senescing transplanted fields into unharvested fields, resulting in higher IYSV levels in directseeded fields. To describe the temporal dynamics of IYSV and its vector in onion fields, onion thrips and IYSV levels were monitored in six direct-seeded and six transplanted fields in 2007 and 2008. More thrips were found in transplanted fields early in the season, but direct-seeded fields had higher levels of IYSV at the end of the season. Levels of IYSV remained low, <7%, throughout June and July, and increased dramatically in August. Thrips densities late in the season were a better predictor of final IYSV levels than early thrips densities. Consequently, the time onions are harvested may be more important in determining final IYSV levels than how onions are planted, and that emigration of viruliferous thrips from harvested fields may be increasing the primary spread of IYSV in late-harvested fields. Managing onion thrips populations before harvest and manipulating the spatial arrangement of fields based on harvest date could mitigate the spread of IYSV.

# Research article: Effect of reflective mulches and plant covers in controlling thrips in floricultural crops

**Published:** 2010-10-01

**Authors:** Julie P. Newman, Karen F. Robb

**Affiliations:** ^1^ University of California Cooperative Extension, 669 County Square Drive, Ventura, CA, 93003-5401, USA; ^2^ Target Specialty Products, 2478 N Sunnyside Ave, Fresno, CA 93727, USA

Some insects use UV light for flight navigation and UV light reflectance patterns for recognizing host plants. Placing materials that reflect UV light on the ground or above plants may interfere with the ability of certain pests to locate and land on plants. Thrips are a major pest of floricultural crops, especially western flower thrips, *Frankliniella occidentalis* (Pergande), which is also a vector of tospoviruses that cause serious crop diseases. Our objective was to evaluate the effects of reflective ground covers and plant covers in reducing thrips populations in commercial crops of field-grown cut chrysanthemums (*Chrysanthemum morifolium*) and goldenrod (*Solidago* ‘Yellow Submarine’), and in outdoor container roses (*Rosa x hybrida*). In the first experiment, plots were established in chrysanthemums and goldenrod at two commercial nursery sites in California, USA. Treatments were reflective ground mulch, reflective cover, a combination of ground mulch with plant cover, and control(s). Sticky traps and plant samples in plot center rows were examined weekly, and all thrips were counted. Additional measurements were light, soil temperature, plant stem weights, and stem length. A rating scale was used to evaluate crop quality. In the second experiment at another California nursery site, a reflective cover was evaluated in outdoor container roses using insect counts from traps and plants samples. Significant differences in the adult thrips populations were observed in all crops, with the two plant cover treatments providing the best protection. Reflective ground mulches alone protected crops initially, but effectiveness was later reduced when the plant canopy covered the mulch. There were significant light and soil temperature differences in the chrysanthemum plots, with the treatments containing reflective covers reducing overall light levels and ground mulches increasing soil temperature. Chrysanthemums grown under the reflective coverings were of better quality, and stem length was increased in both chrysanthemums and goldenrod.

# Research article: Do attractants lure thrips into greenhouses?

**Published:** 2010-10-01

**Authors:** Mette Nielsen, D.E. Hartnett, R.I.F. Pedley, D.A.J. Teulon

**Affiliations:** ^1^ The New Zealand Institute for Plant & Food Research Limited, Private Bag 4704, Christchurch 8140, New Zealand; ^2^ The New Zealand Institute for Plant & Food Research Limited, Private Bag 92169, Auckland 1142, New Zealand

Semiochemical-based tools, such as thrips lures, are becoming more commonly used for monitoring thrips pests in greenhouses. An important concern is that thrips from outdoor populations might be lured into the greenhouses in greater numbers when the lures are being used. To investigate this issue, intensive trapping of thrips (*Thrips* and *Frankliniella*) was carried out in a capsicum (*Capsicum annuum*) greenhouse crop in New Zealand in the summer months of February and March in 2007 and in 2008. Blue sticky traps were positioned above the crop canopy for 4 days per week for 3 weeks per year: 2 days with and 2 days without prototype alleochemical lures. Traps without lures were also placed at the greenhouse vents above the trapping areas. Additionally, thrips were sampled from plant flower heads and shoots and a suction trap (2 m above the ground) located in the trapping area. Numbers of thrips from sticky traps, the suction trap and plant samples were counted and identified to species level. There were large variations in both the numbers of thrips caught and the species composition for all trapping methods between weeks and years. Lure-baited traps caught overall more thrips than non-baited traps for both years. Numbers of thrips caught on the traps at the vents reflected the number of thrips caught in the crop traps, irrespective of whether they were baited or not. No consistent pattern of thrips was observed with the presence or absence of lures in the greenhouse. Numbers of thrips estimated from plants were either the same or higher after a period without lures in the greenhouse than after a period with lures. There was no strong indication that the presence of lures increased the number of thrips or changed the species composition of the thrips populations in the greenhouse in either year.

# Research article: A method to study thrips' feeding habits

**Published:** 2010-10-01

**Authors:** C. Nischwitz, Stephen W. Mullis, K. Lewis, R.D. Gitaitis

**Affiliations:** ^1^ Department of Plant Sciences, Division of Plant Pathology and Microbiology, University of Arizona, Tucson, AZ, 85721-0036, USA; ^2^ Department of Plant Pathology, University of Georgia, Tifton, GA, 31793-0748, USA

*Tomato spotted wilt virus* (TSWV) continues to be a major problem in tobacco (*Nicotiana tabacum*) and peanut (*Arachis hypogaea*) production in Georgia. In tobacco, current management strategies include using compounds that induce systemic acquired resistance (SAR) and insecticides to control thrips vectors. Thrips have to acquire the virus before they can infect tobacco plants in the spring. If the weed host reservoirs in the vicinity of tobacco fields can be identified, they could be targeted for elimination to reduce inoculum. There are over 2,000 hosts of TSWV, many of them non-symptomatic weeds, but only a few may serve as a source of inoculum for TSWV epidemics in tobacco. To determine the last food source of thrips flying into tobacco fields, individual *Frankliniella occidentalis* were collected from the surface of a white vehicle positioned at the edge of tobacco fields using a modified vacuum filtration system. Immediately after collection, containers were put on ice to immobilize thrips and slow metabolic activity until DNA extraction. Upon return to the lab (∼15 min.), collection containers were placed in a -80C freezer. Total DNA was extracted and universal primers for the ITS region of nrDNA were used for PCR and the resulting products were sequenced. Sequences subjected to a BLAST search in GenBank matched ragweed(*Ambrosia artemisiifolia*)and tomato (*Lycopersicon esculentum*) among others. A time-course study, in which thrips were starved but allowed to remain active for varying time periods prior to freezing, indicated their food sources could be identified up to 15 hours after feeding but not after 24 hours. Although adult thrips cannot acquire the virus, this method has the potential of identifying plants that *F. occidentalis* not only prefers to feed upon but also reproduce.

# Research article: Epidemiology of *Iris yellow spot virus* in Georgia, USA

**Published:** 2010-10-01

**Authors:** C. Nischwitz, Stephen W. Mullis, R. Srinivasan, R.D. Gitaitis

**Affiliations:** ^1^ Department of Plant Sciences, Division of Plant Pathology and Microbiology, University of Arizona, Tucson, AZ, 85721-0036, USA; ^2^ Department of Plant Pathology, University of Georgia, Tifton, GA, 31793-0748, USA; ^3^ Department of Entomology, University of Georgia, Tifton, 31793-0748, GA, USA

*Iris yellow spot virus* (IYSV) was discovered in Georgia in 2003. The virus has a limited host range and onion, the only known host commercially grown in Georgia, is only produced in a regulated area [Vidalia onion zone (VOZ)]. Previously, spiny sowthistle (*Sonchus asper*) was confirmed as a host. Consequently, spiny sowthistle was used to conduct a survey across Georgia to determine the distribution of IYSV within and outside the VOZ. The survey was conducted 2007–2009 within the VOZ and along four line transects from the center of the zone or parallel to it. Samples were collected from 42 counties and tested with commercial ELISA kits. In 2007, IYSV-infected sowthistles were found 333 km northwest of the VOZ, as well as near the experiment station to the west in Tifton. The survey showed that 36% of all counties sampled had at least one sowthistle plant test positive for IYSV in 2007, which increased to over 50% of the counties having a sowthistle plant test positive by 2009. Similar distributions were observed in all three years, except additional sites tested positive over time, especially in the western sites where IYSV- infected sowthistles were detected up to 265 km from the VOZ. However, no infected sowthistles were detected to the south and east of the VOZ in all three years, despite the fact that these five counties were adjacent to the border of the VOZ. Attempts to confirm ELISA results were inconclusive using regular reverse transcriptase polymerase chain reaction (RT-PCR). However, using the same primers for N gene segments, high fidelity (HF) RT-PCR could routinely confirm ELISA results. Ten IYSV sequences were obtained from various locations within the state. Subjected to a BLAST search against sequences in GenBank, all sequences most closely matched strains previously reported from Georgia or Peru.

# Research article: Thrips competition: is *Frankliniella occidentalis* really the big bully on the corner block?

**Published:** 2010-10-01

**Authors:** T.D. Northfield, Dean R. Paini

**Affiliations:** ^1^ Department of Entomology, Washington State University, Pullman, WA 99164, USA; ^2^ CRC — National Plant Biosecurity, Entomology Division, CSIRO, Clunies Ross St, Acton, ACT 2601, Australia

High competitive ability is believed to be an important characteristic of invasive species. Many animal studies have compared the competitive ability of invasive species with a native species that is being displaced, but few studies have evaluated systems where an invasive species has failed to establish itself. The thrips species *F. occidentalis* is a highly invasive crop pest that has spread from its original range (the western states of the US) to a worldwide distribution. Despite this, *F. occidentalis* is largely absent or in low numbers in southern Florida where the native *F. bispinosa* dominates and in the eastern states of the US, where *F. tritici* dominates. It is possible that both *F. bispinosa* and *F. tritici* are competitively excluding *F. occidentalis* from this region. We present experimental evidence that *F. occidentalis* is competitively superior to *F. bispinosa* yet competitively inferior to *F. tritici*. We discuss the implications for such competitive interactions for *F. occidentalis* distribution and the implications that climate change could have on TSWV transmission by *F. occidentalis*.

# Research article: Thrips species composition and abundance on French beans, associated crops and weed species in Kenya

**Published:** 2010-10-01

**Authors:** Johnson O. Nyasani, R. Meyhöfer, Subramanian Sevgan, H-M. Poehling

**Affiliations:** ^1^ Kenya Agricultural Research Institute, P O Box 27-60100 Embu, Kenya; ^2^ Leibniz Universität Hannover, Institute of Plant Protection and Plant Diseases, Herrenhäuser Strasse 2, 30419 Hannover, Germany; ^3^ International Centre of Insect Physiology and Ecology, P O Box 30772-00100 Nairobi, Kenya

Thrips are among the major pests of French beans in Kenya. This study aimed at determining the thrips species composition and abundance on French beans and intercrops such as Irish potato, baby corn, sunflower, and associated weeds. Field experiments were established at Kenya Agricultural Research Institute, Embu, Kenya from January to May 2009 in a randomised complete block design. Source fields around the experimental plots were planted in NS and EW directions four weeks before the main crop. Ten plants from each plot were randomly sampled and thrips collected by beating them against a white tray. Additionally three leaves and flowers were randomly sampled from ten plants for thrips collection. The thrips were processed and identified using the LucID key Pest thrips of the world. The results show that thrips population in different crops increased from 1–3 thrips/plant at 2–6 leaf stage to 21-fold at flowering. At crop senescence, the population declined except for baby corn where more thrips were found on the silk. The thrips species *Frankliniella schultzei, Megalurothrips sjostedti, Hydatothrips adolfifriderici* and *F. occidentalis* (41.1, 34.3, 17.7, 6.4% respectively) were commonly found on French bean as compared to Irish potato and baby corn, which supported mainly *F. schultzei*, 92.0%, and *F. williamsi,* 87.9%, respectively. In contrast sunflower hosted both *F. occidentalis* (39.5%) and *F. schultzei* (30.2%). Among the weed species *Amaranthus sp., Nicandra physaloides*, and *Galinsoga parviflora* supported mainly *F. occidentalis*, while *Datura stramonium* and *Portulaca oleracea* supported mainly *F. schultzei.* In conclusion it is likely that thrips problems on French bean crops are caused by a complex of at least four different species. Although the specific role of the different thrips species is unclear, they show a preference for certain crops and weeds, which in turn might act as a source for new infestations.

# Research article: The economic importance and impact of western flower thrips (*Frankliniella occidentalis* Pergande) and thrips detection on commodities at US ports of entry

**Published:** 2010-10-01

**Authors:** Cheryle A. O'Donnell

**Affiliations:** United States Department of Agriculture, Animal Plant Health Inspection Services

A historical review of the economic impact and research of western flower thrips (*Frankliniella occidentalis* Pergande) is summarised. The focus of historical research and the energy spent to prevent and management western flower thrips and tospovirus is highlighted. A review of investments on reducing importation and exclusion of this cosmopolitan thrips is also investigated. What do ports of entry focus on and what are their capabilities for detecting thrips on imported commodities? Where has our energy been focused? Lastly, we visit the present research, the current cost of management and outline the focus for the future. Are we heading in the right direction?

# Research article: Colour polymorphism and genetic variation of Western flower thrips in California

**Published:** 2010-10-01

**Authors:** Cheryle A. O'Donnell

**Affiliations:** United States Department of Agriculture, Animal Plant Health Inspection Services, Plant Protection and Quarantine 9 North Grand Avenue, Nogales AZ 85621, USA

Western flower thrips, *Frankliniella occidentalis* Pergande, is a well known pest in California and is polymorphic for colour, with populations varying from nearly black to pale yellow. This type of colour polymorphism has been noted throughout the world. Several hypotheses have been proposed for why these colour morphs occur, i.e. variation in temperature or host plant, or genetic differences within the species. A comparison of sequence data from nuclear genes, ITS and 28S, and mitochondrial genes, COI and 16S, have been analysed to evaluate the genetic variation among western flower thrips from California. In addition, a genetic comparison was analysed from WFT specimens from Europe and several states in the United States as well as other *Frankliniella* sp. thought to be closely related to WFT. A dendrogram revealing the relationship between colour polymorphism and genetic similarity in western flower thrips and outgroup taxa is presented.

# Research article: Evaluation of *Tomato spotted wilt virus* resistant tomato cultivars in southeastern USA

**Published:** 2010-10-01

**Authors:** Stephen M. Olson, D. Riley, J.W. Scott

**Affiliations:** ^1^ University of Flonda/IFAS, NFREC, Quincy, FF 32351; ^2^ University of Georgia, Athens, GA 30602; ^3^ University of Flonda/IFAS, GCREC, Wimauma, FF, USA

Since *Tomato spotted wilt virus* (TSW) was first isolated in the southeastern USA in 1986, it has become the number one disease problem of tomatoes in this region especially in home gardens and with small growers. Management has been through metalized mulches, selected insecticides, systemic acquired resistant materials and newer resistant cultivars. Resistance with the Sw-5 gene has been around for about 12 years but early hybrids had production or quality problems. Recently many new hybrids have been released that have superior yield and fruit quality. There has also recently been an increase in a strain or strains of *Tomato spotted wilt virus* that can overcome the Sw-5 gene. These trials were conducted to evaluate new large round fresh-market tomato hybrids that contain either the Sw-5 gene or Sw-7 gene (new source of TSW resistance). In the spring of 2009, twenty seven hybrids were evaluated at the NFREC, Quincy and Tifton, GA research centers. Trials were replicated 4 times at each location. Of the 27 hybrids evaluated, 21 had the Sw-5 gene, 2 had the Sw-7 gene, 1 had both Sw-5 and Sw-7 genes and 3 had no TSW resistance (2 were resistant to Tomato yellow leaf curl). Information will be presented on the performance of these hybrids as to yield and incidence of TSW. Additional information will be provided on the Sw-7 gene as to source, selection and early performance of hybrids constructed with it present.

# Research article: IPM strategies for reducing the impact of *Iris yellow spot virus* epidemics in onion

**Published:** 2010-10-01

**Authors:** Hanu R. Pappu

**Affiliations:** Department of Plant Pathology, Washington State University, Pullman, WA, USA

*Iris yellow spot virus* (IYSV) is the most economically important viral pathogen of onion bulb and seed crops in the Pacific Northwest (PNW) of the United States. New reports of IYSV have emerged in other parts of the country as well from other countries. Complete crop loss due to IYSV was reported in PNW in some years. IYSV is transmitted by onion thrips (*Thrips tabaci* Lindeman) and there is no evidence of seed transmission. Hence, thrips play a critical role in IYSV epidemiology. While control options to reduce the disease impact are limited, progress has been made in understanding some of the factors that seem to contribute to the outbreaks. Lack of resistance to IYSV in commercial onion cultivars combined with prevalence of high vector populations due to limited thrips control options and the availability of abundant virus inoculum could be leading to the severe disease outbreaks observed in recent years. In some onion producing regions of the country, the overlapping seasons of the annual bulb and the biannual seed crops provide the green bridge for both the virus and the thrips vector. At present, growers are advised to avoid crop stress related to soil fertility, irrigation, and observe sanitation. Field evaluation of onion varieties under natural conditions in PNW has identified some with field tolerance. Continued evaluations of breeding material are in progress in different parts of the country. Identification of risk factors that influence final disease incidence need to be identified and an IPM strategy based on the risk factors could potentially reduce the impact of IYSV. A similar strategy based on a risk index has been highly successful in case of reducing the impact of *Tomato spotted wilt virus* in peanut in southeastern US. Component tactics for IPM for thrips and IYSV in onion should address aspects of virus avoidance (isolation of *Allium* seed crops and winter bulb crops from summer onion bulb crops, management of culls and volunteer onions, virus-free transplants, manipulation of the planting date, identification and management of non-*Allium* hosts of IYSV, avoiding stress (soil moisture, salts), identification of less susceptible or tolerant varieties and sources of IYSV resistance, development of resistant varieties, and sustainable thrips management strategies.

# Research article: Epidemiology of *Iris yellow spot virus*: new hosts and seasonal dynamics of thrips vectors

**Published:** 2010-10-01

**Authors:** Hanu R. Pappu, S. Bag, S. Rondon

**Affiliations:** Department of Plant Pathology, PO Box 646430, Washington State University, Pullman, WA 99164, USA

*Iris yellow spot virus* (IYSV), a member of the genus *Tospovirus* and family *Bunyaviridae*, causes an economically important disease in bulb and seed onion crops in several parts of the USA. The Pacific Northwest of the USA has been particularly affected by serious outbreaks of this virus. Onion thrips are the only known insect vector for IYSV. Developing tools to monitor and manage viruliferous onion thrips could provide an effective management tool to manage this disease. An ELISA-based assay was developed to identify onion thrips that could be potential transmitters of IYSV. As part of a multiyear study, beginning in 2008, onion thrips were monitored in two field plots at the Oregon State University's Hermiston Agricultural Research and Extension Center, on a weekly basis using the fullplant count technique. Each week, at least 20 onion thrips were collected from each field and from each sampling site. Preliminary data showed that onion fields planted next to overwintering onions, a potential source of onion thrips for the following season, did not increase the mean number of onion thrips per plant per week in the field planted adjacent to it. Using antiserum specific to the NSs protein of IYSV, thrips were tested using ELISA to identify potential virus transmitters (viruliferous thrips). The seasonal dynamics of viruliferous thrips could be useful in refining thrips management practices for reducing the impact of IYSV in onion.

# Research article: *Tomato spotted wilt virus* infection of rhubarb

**Published:** 2010-10-01

**Authors:** Kathy S. Parmenter, J.E. Thomas

**Affiliations:** Queensland Primary Industries and Fisheries, Department of Employment, Economic Development & Innovation, Indooroopilly, Queensland 4068, Australia

During a national virus survey to investigate rhubarb decline disease, rhubarb (*Rheum rhaponicum*) samples, with symptoms including chlorotic spots, red ringspots, or a chlorotic mosaic along central leaf veins, were collected from a crop near Stanthorpe, Queensland. Tospovirus-like particles, but no other virus-like particles, were observed by electron-microscopy of sap, and infection by *Tomato spotted wilt virus* (TSWV) was demonstrated by ELISA. This was confirmed by a TSWV-specific PCR. Samples were negative by ELISA for other viruses which commonly infect rhubarb. Further survey results identified TSWV infection of rhubarb crops in the Sydney Basin, NSW, and on the Mornington Peninsula, Victoria. This appears to be the first record worldwide of TSWV infecting rhubarb.

# Research article: Breeding for tospovirus resistance in capsicum and tomato in Australia

**Published:** 2010-10-01

**Authors:** Denis M. Persley, D. McGrath, M. Sharman, I.O Walker

**Affiliations:** ^1^ Queensland Primary Industries and Fisheries (QPIF), 80 Meiers Road, Indooroopilly, Queensland 4068; ^2^ QPIF, Gatton Research Station, FMB 7 MS 437, Gatton, Queensland 4343; ^3^ QPIF, Bowen Research Station, PO Box 538, Bowen, Queensland 4805, Australia

Capsicum chlorosis virus (CaCV) is a member of the *Watermelon silver mottle virus* group of tospoviruses (McMichael et al. 2002) and infects capsicum and tomato. In Australia, the virus was first identified in Queensland and is now endemic in the major capsicum production areas of the State. The most significant damage is in capsicum crops as tomato is rarely severely infected in open-field conditions. Resistance to CaCV was identified and characterised in PI 290972 of *Capsicum chinense* and then developed through several cycles of conventional backcrossing in advanced bell capsicum breeding lines. The resistance to CaCV is not effective against *Tomato spotted wilt virus* and segregates as a dominant gene, independently of the *Tsw* gene for TSWV resistance. A series of CaCV-resistant bell capsicum breeding lines has been released from which hybrid cultivars incorporating both CaCV and TSWV resistances will be developed for commercial use. There is no effective CaCV resistance available in commercial tomato cultivars. By contrast, the *Sw-5* gene for TSWV resistance is highly effective and has been transferred to a broad range of international cultivars. Unlike *Tsw* in capsicum, *Sw-5* in tomato is stable and widely effective in Australia, and we are using marker assisted selection for *Sw-5* in the development of indeterminate trellis cultivars. In addition to TSWV resistance, we are applying markers to the transfer of resistance genes for *Tomato* *yellow leaf curl virus*, Fusarium wilt and nematodes. Markers for powdery mildew resistance are under development in collaborative research and will be included later in the wider program. Our strategy is to introduce *Sw-5* to a comprehensive package of multiple-disease resistance in well-adapted elite breeding lines and hybrid genotypes.

## References

[bibr01] McMichael L, Persley DM, Thomas J (2002). A new tospovirus serogroup IV species infecting capsicum and tomato in Queensland Australia.. *Australasian Plant Pathology*.

# Research article: The importance of reporting suspect exotic or emergency plant pests to your state Department of Primary Industries

**Published:** 2010-10-01

**Authors:** Sophie Peterson, F.J. Macbeth

**Affiliations:** ^1^ Plant Health Australia, 5/4 Phipps Close, Deakin, 2600, ACF; ^2^ Australian Government Department of Agriculture, Fisheries and Forestry, GPO Box 858, Canberra, 2601, ACF, Australia

The Emergency Plant Pest Response Deed (EPPRD) is a formal legally binding agreement between Plant Health Australia (PHA), the Australian Government, all State and Territory Governments and plant industry signatories covering the management and funding of eradication responses to Emergency Plant Pest (EPP) Incidents. Plant Health Australia is the Custodian of the EPPRD. For an eradication response to be agreed it must be both technically feasible and cost beneficial to eradicate the pest. As such, early reporting of suspect emergency plant pests is a critical step in the process. The longer it takes for a suspected EPP to be reported, the more time the pest has to become established and widespread. This increases the costs of containment, control and eradication measures, reduces the technical feasibility and therefore reduces the likelihood of success of eradication. It is important that diagnosticians and researchers understand their responsibility, not only a moral obligation to protect Australian agriculture and horticulture but this legal obligation that now exists for jurisdictions and their personnel. Personnel of government agricultural agencies need to report a ‘reasonably held suspicion’ of an exotic pest to their jurisdiction's Chief Plant Health Manager directly or via the Exotic Plant Pest Hotline (1800 084 881). Not only does the EPPRD provide an obligation to report a ‘reasonably held suspicion’ but there is the potential for cost sharing of actions taken to be rejected if it is deemed that there has been a failure to report in a timely manner.

Many significant plant pests are cryptic and not readily visible. Red Imported Fire Ant is estimated to have been present for as many as five years before detection, likewise European House Borer may have been present for as long as 50 years before detection. Both of these pests would have cost considerably less to eradicate if they had been detected and reported within the first few generations.

# Research article: Predator-prey interactions between *Orius insidiosus* and flower thrips

**Published:** 2010-10-01

**Authors:** Stuart R. Reitz

**Affiliations:** SDA-ARS, Center for Medical, Agricultural and Veterinary Entomology, Tallahassee, FL, USA

The great debates generated in Australia some 50 years ago regarding the relative merits of density dependent versus density independent forces in population dynamics were both reiterations of earlier ecological debates and precursors of succeeding ones. Perhaps, as has been recently emphasised, the question shouldn't be if populations are regulated, but the extent to which populations may be regulated. From the perspective of managing pest thrips, this becomes an important distinction as thrips populations may in fact show strong density dependence, but still occur at levels that are higher than economically tolerable. The view that natural enemies, or other density dependent factors, cannot suppress pest thrips populations below economically injurious levels has recently been challenged in certain agroecosystems. This presentation will focus on interactions between the anthocorid predator *Orius insidiosus* (Hemiptera: Anthocoridae) and different species of *Frankliniella* flower thrips, including how species specific traits affect interactions. From a population ecology perspective, this predator shows the ability to regulate flower thrips populations, even in heterogeneous environments. From a pest management perspective, naturally-occurring populations of *Orius insidiosus* have the capacity to suppress pest flower thrips populations below economically damaging levels in certain agroecosystems. Factors affecting the ability of this predator to be an effective biological control agent of flower thrips will be discussed.

# Research article: Pine pollen deposition effects on reproduction of *Frankliniella* spp.

**Published:** 2010-10-01

**Authors:** David G. Riley

**Affiliations:** University of Georgia, Tifton Campus, Tifton, GA 31793, USA

Tree pollen, especially *Pinus* spp., is shed in large quantities every spring in North America. Pine pollen deposition onto leaves has been found to significantly increase various reproductive behaviours of thrips vectors of *Tomato spotted wilt virus* (TSWV) on various crops. In previous studies, slash pine pollen (*Pinus elliottii* Engelm.) increased the oviposition rate on foliage three-fold for *Frankliniella occidentalis* (western flower thrips) and two-fold for *Frankliniella fusca* (tobacco thrips) averaged over two crops, peanut and tomato. The impact of pollen on the rate of oviposition by thrips is important because the addition of pine pollen to TSWV infected plant foliage has been shown to increase the percentage of infected *F. fusca* after one generation. In the current study, pine pollen (*Pinus spp*.) was monitored using a Burkard air sampler and pollen collection sheets to quantify pollen dispersal and deposition during the spring of 2005–2008. Yellow sticky cards were used to monitor thrips (Thysanoptera: Thripidae) dispersal near the pollen collection station. Positive correlations were detected over the four-year period between thrips on traps and pollen events two weeks prior to the trap counts. In addition, leaf tissue from various crops with and without pine pollen was used to bioassay thrips for effects of pollen deposition on oviposition and net reproduction. In leaf cage studies in tobacco the addition of slash pine pollen increase the number of off spring four-fold for *F. fusca* and 20-fold for *F. occidentalis*. As in previous studies, pine pollen had a greater impact on flower thrips (*F. occidentalis*) than foliage thrips (*F. fusca*) reproduction. Using a new micro cage technique with onion seedlings, the addition of pine pollen significantly increased net reproduction of *F. fusca* by 340%. The micro cage technique provided an efficient method for assessing thrips life table parameters.

# Research article: A reduced-risk system for managing thrips and TSWV in tomato and pepper

**Published:** 2010-10-01

**Authors:** David Riley, George Kennedy, James Walgenbach, Stephen M. Olson, J. Powell Smith, Ron Gitaitis, James Moyer, Greg Fonsah, Jay W. Scott, Alton ‘Stormy’ Sparks, Mark Abney, David Fangston, Frank Louws, Christopher Gunter

**Affiliations:** ^1^ University of Georgia, Athens, GA 30602, USA; ^2^ North Carolina State University, Raleigh, NC 27695, USA; ^3^ University of Florida, Gainesville, FF 32611, USA; ^4^ Clemson University, Clemson, SC 29631, USA

Since 1986, thrips-vectored *Tomato spotted wilt virus* (TSWV) has become one of the major causes of diseases of crops in the southeastern USA, causing as much as $100 million in damage annually. Two important crops at-risk from TSWV are fresh tomato and pepper. Production of these crops in FL, GA, NC, and SC represents 54% of the USA annual production ($1.3 billion) and the majority of acreage affected by TSWV. TSWV is transmitted in the southeast primarily by tobacco thrips, *Frankliniella fusca*, and western flower thrips, *Frankliniella occidentalis*. Management of TSWV in tomato and pepper is difficult and involves the use of multiple preventive tactics, including TSWV-resistant plant cultivars, reflective mulch, chemical treatments, and weed management. No single tactic provides 100% control and all must be applied prior to or during early disease spread to be effective. Thus, an integrated multi-tactic management approach is needed. The goal of this project is to develop a reduced-risk management system for thrips and TSWV in tomato and pepper in the Southeast. The objectives are to: 1) Optimise use of available TSWV management options and define their effectiveness, costs and benefits when used alone and in combination. 2) Refine and extend weather-based models for predicting the risk of TSWV spread into crops in spring. 3) Develop a risk based decision guide for growers to evaluate the need for and the optimum combination of TSWV management tactics. 4) Facilitate implementation of reduced risk thrips and TSWV management in tomato and pepper through out-reach programs. In the fall of 2008, a survey was conducted to assess the status of TSWV management in the southeast. In the spring of 2009, multiple field experiments were conducted in GA, NC, SC and FL to evaluate most of the best management practices currently available and validate a method for TSWV risk prediction.

# Research article: WFT: WTF? (Western flower thrips: what, two *Frankliniella*?)

**Published:** 2010-10-01

**Authors:** Paul F. Rugman-Jones, M.S. Hoddle, R. Stouthamer

**Affiliations:** Department of Entomology, University of California, Riverside, CA 92521, USA

Over the last 30 years, the western flower thrips (WFT), *Frankliniella occidentalis* (Pergande) (Thysanoptera: Thripidae), has become a serious worldwide pest of many agricultural and horticultural crops. WFT is highly polyphagous and causes direct damage to fruits and flowers, and also acts as a major vector of tospoviruses, most notably *Tomato spotted wilt virus* (TSWV). Native to an area west of the Rocky Mountains (USA), WFT is extremely widespread throughout the state of California, and occurs across a huge variety of vegetation types from the pacific coast to the interior mountain ranges at elevations up to 9,000 ft. The morphology of WFT is highly variable, a fact most readily exemplified by the existence of three cooccurring colour-morphs: pale, intermediate, and dark. Colour has been shown to have a simple mendelian genetic basis, and the colour-morphs appear to be equally efficient at vectoring TSWV. However, colour is also influenced by abiotic factors such as temperature. Significant variation between WFT populations has also been shown in pest status and resistance to insecticides, with so called ‘glasshouse strains’ being more resistant than native field populations in California and the ‘lupin strain’ in New Zealand. The high degree of polyphagy, and levels of morphological and molecular variation, suggest that what we currently recognise as *F. occidentalis* may in fact be a complex of several cryptic species. Here, we consider variation in *F. occidentalis* at the genetic level, examining DNA sequence data from both nuclear and mitochondrial genes. Sequence data from WFT specimens collected throughout California suggest the existence of two sympatric, and reproductively isolated, genetic ‘types’. This finding is discussed with respect to currently recognised ‘strains’, the evolution of the two sympatric types, and whether the genetic ‘types’ represent different species with different pest potential.

# Research article: Investigations of leaf chemistry and evasion of polyphagous species of thrips

**Published:** 2010-10-01

**Authors:** Alison Scott-Brown, Nigel C. Veitch, Monique S.J. Simmonds

**Affiliations:** Jodrell Faboratory, Royal Botanic Gardens, Kew, Surrey, FW9 3AB, UK

The large display glasshouses at Kew contain up to 4,000 different species of plants, several of which are listed as rare and endangered in their natural habitats. A large proportion of these unique plant specimens are subject to attack from polyphagous thrips species which are difficult to control due to the restrictions on chemical usage and plant-host preferences displayed by natural enemies applied to this environment. However, it has been observed that distributed among the numerous thrips ‘hosts’ are plants which consistently avoid invasion by thrips. This prompted a detailed study of the interactions of two polyphagous thrips species; *Frankliniella occidentalis* (Pergande) and *Heliothrips* *haemorrhoidalis* (Bouché) and several species of plants recorded as ‘non-hosts’. The foliage of the non-hosts was found to have toxic and/or deterrent properties against thrips and compounds known to be active against insects were detected in several of the plant species investigated. Crude leaf extracts of *Sclerochiton harveyanus* Nees. (Acanthaceae) demonstrated the highest levels of activity against *F. occidentalis*. These extracts contained iridoids, including two new compounds of this group which were characterised using spectroscopic methods. Bioassays concluded that the mortality of *F. occidentalis* was increased on exposure to the iridoid glycosides and residues of these compounds tended to deter *H. haemorrhoidalis* from treated ‘host’ leafsurfaces. Providing further insights into the mechanisms involved in plant resistance to thrips can serve to increase the possibilities of controlling thrips in environments where the use of current control techniques is limited.

# Research article: Computer-based identification key for pest thrips and tospoviruses by use of LucID 3.4, ITS-RFLP and low-density BioChip technology

**Published:** 2010-10-01

**Authors:** Ingeborg Schuetz, Antje Breitenstein, Gerald Moritz

**Affiliations:** ^1^ Martin Luther University of Halle and Wittenberg, Institute for Biology, Group Developmental Biology, Halle, Germany; ^2^ Becit GmbH, Wolfin-Bitterfeld, Germany

Thrips account for billions of dollars worth of agricultural losses every year. Besides being a pest thrips itself, some species spread tospoviruses due to their feeding behaviour. Identification of pest thrips and transmitted viruses as early as possible is important to implement the appropriate pest control measures. We present a computer-aided identification key based on LucID 3.4 for pest thrips using typical character states visible under bright field or phase contrast microscope. More than 1000 computerised microscopic photomicrographs and fact sheets for each species are included in the identification key. We also established a web-accessible data base for molecular identification (ITS-RFLP) of thrips of any developmental stage (adult, prepupa/pupa, larva, egg). After DNA-extraction from insect or leaf material, PCR, digestion with restriction enzymes and gel electrophoresis, the resulting fragment size can be compared online with our php-data base to identify the species.

We are developing also in a next step a low density bio chip to identify pest thrips of any developmental stage. The new biochip will identify both thrips and tospoviruses transmitted by the insect simultaneously. The newly developed technique will be small, robust and cheap enough to be used both in lab and field. All three parts will be integrated into one computer-based identification tool running on most computer platforms (MS Windows 98 or higher, Linux, Sun, MacOS X).

We thank the German Ministry of Agriculture for financial support.

# Research article: Altitudinal differences in abundance and diversity of thrips on tomatoes (*Lycopersicon esculentum* Mill.) in East Africa

**Published:** 2010-10-01

**Authors:** Subramanian Sevgan, A. Mayamba, B. Muia, P. Sseruwagi, J. Ndunguru, F. Fred, M. Waiganjo, M.M. Abang, G.B. Montz

**Affiliations:** ^1^ International Centre of Insect Physiology and Ecology, Nairobi, Kenya,; ^2^ National Crops Resources Research Institute (NaCRRI), Namulonge, Uganda; ^3^ Mikocheni Agricultural Research Institute, Dar es Salaam, Tanzania; ^4^ Kenya Agricultural Research Institute, Fhika, Kenya; ^5^ AVRDC-Fhe World Vegetable Center, Arusha, Tanzania; ^6^ Martin Futher University, Halle, Germany

Tomatoes are grown extensively by smallholders across East Africa as a high-value horticultural crop for the local market. Its production is seriously constrained by several arthropod pests and diseases. Thrips are among the most frequently reported pests of tomato in the region and they also vector tospovirus diseases. It is of crucial importance to understand the diversity of thrips, especially vectors of *Tomato spotted wilt virus*, for their effective management. Hence a region wide survey on thrips fauna associated with tomato in over 60 locations was undertaken across Kenya, Uganda and Tanzania. Representative thrips samples from the field were processed and identified using the LucID key, Pest thrips of the world (2004) and the key by Palmer et al. (1992), and a reference collection has been established. More than 12 thrips species occurred on tomato including *Ceratothripoides brunneus* (Bagnall, 1918)., invasive western flower thrips (WFT), *Frankliniella occidentalis* (Pergande, 1895), *Frankliniella schultzei* (Trybom, 1910) and *Thrips tabaci* (Lindeman 1988), which were the dominant species. The average thrips density per plant was more in mid altitude regions (600 – 1800m) (11.2±2.5) in comparison to the high altitude regions (>1800m) (2.5±0.7). The diversity of thrips collected on tomato varied considerably with the altitudinal gradient with more thrips species in the mid altitude regions and *C. brunneus* was the dominant species (82.7%, n=479). In the highlands the diversity was less with the WFT being the dominant species (36.67%, n=32) and *C. brunneus*. was absent beyond 2000m. The invasive WFT was found to be wide spread in Kenya on different crops (16%, n=2171), while it was not prevalent in Uganda (0.19%, n=523) and Tanzania (1.38%, n=145). The relevance of these findings to the management of thrips and tospoviruses on tomato in East Africa are discussed.

# Research article: Thysanoptera inhabiting native terrestrial orchids in southeastern USA

**Published:** 2010-10-01

**Authors:** Jyotsna Sharma, J. Funderburk, L. Mound

**Affiliations:** ^1^ Texas Tech University, Fubbock, Texas 79409, USA; ^2^ University of Florida-NFREC, Quincy, Florida 32351, USA; ^3^ CSIRO Entomology, PO Box 1700, Canberra ACT 2601, Australia

A survey was conducted to identify the thrips species associated with native terrestrial orchids in southeastern USA. Above-ground plant structures were sampled when each orchid species was flowering yielding adults of polyphagous species of the common flower thrips *Frankliniella tritici* (Fitch), *Frankliniella fusca* (Hinds), and *Microcephalothrips abdominalis* (Crawford). There is little indication that the orchids were reproductive hosts of these species. Other species of thrips that are more host-specific to orchids were collected and included adults of the orchid-living species *Aurantothrips orchidaceus* (Bagnall) and *Pseudothrips beckhami* Beshear and Howell. The presence of larvae of *P. beckhami* indicated the suitability of at least some orchid species as reproductive hosts. The adults of two predatory species in the Family Phlaeothripidae also were collected, *Leptothrips mali* (Fitch) and *Karnyothrips melaleucus* (Bagnall).

# Research article: A new tospovirus species from Thailand

**Published:** 2010-10-01

**Authors:** Murray Sharman, P. Chiemsombat, P.R. Campbell, D.M. Persley

**Affiliations:** ^1^ Queensland Primary Industries and Fisheries, 80 Meiers Road, Indooroopilly, Queensland 4068; Australia; ^2^ Department of Plant Pathology, Faculty of Agriculture at Khamphaeng Saen, Kasetsart University Khamphaeng Saen Campus, Nakhon Pathom Province, 73140 Thailand

In March 2007 a tomato sample (Isolate-Q1510) displaying fine necrotic rings and spots on the leaves was collected from Ratchburi, Thailand. The sample was negative in Tospo IV ELISA (Agdia reagent set). Partial L-gene was amplified using degenerate tospovirus primers gL3637 and gL4435c (Chu et al. 2001) and the resulting sequence (GenBank accession GQ487713) was most closely related to *Watermelon silver mottle virus* (AF133128) with a nucleotide (nt) identity of 78% over a 752 bp overlap. Degenerate primers were designed and fragments of M-RNA and S-RNA were also amplified from isolate-Q1510. The M-RNA fragment (640 bp) from Isolate-Q1510 had a nt identity of 98 % with the sequence FJ947152, recently published by Seepiban and co-workers who propose the name of Tomato necrotic ringspot virus (TNRV) for this new tospovirus. The next closest match for M-RNA of isolate-Q1510 was a nt identity of 71 % with Peanut bud necrosis virus (PBU42555). The S-RNA fragment amplified from isolate-Q1510 was 635 bp, including 582 nt of the partial N-gene. This had a nt identity of 99 % with TNRV (FJ489600). The next closest match for S-RNA of isolate-Q1510 was a nt identity of 65 % with Groundnut bud necrosis virus (AY871098). The partial M-, and S-RNA sequence of isolate-Q1510 indicate it is Tomato necrotic ringspot virus, a new tospovirus species from Thailand. This is the first report of L-RNA sequence for TNRV. Other isolates of TNRV were also collected from Chiangrai, northern Thailand. Field and experimental host range of TNRV has been investigated.

## References

[bibr02] Chu F.-H., Chao C.-H. (2001). Completion of the genome sequence of *Watermelon silver mottle virus* and utilization of degenerate primers for detecting tospoviruses in five serogroups.. *Phytopathology*.

# Research article: Thrips and seed transmission, and the epidemiology of *Tobacco streak ilarvirus* in Queensland Australia

**Published:** 2010-10-01

**Authors:** Murray Sharman, D.M. Persley, J.E. Thomas

**Affiliations:** ^1^ Queensland Primary Industries and Fisheries, Plant Pathology Building, 80 Meiers Road, Indooroopilly, 4068, Queensland; ^2^ School of Integrative Biology, University of Queensland St. Fucia Campus, Queensland Australia

*Tobacco streak virus* (TSV) is transmitted by thrips and has recently been reported from several important crops in central Queensland Australia, including sunflower, mungbean, chickpea and cotton (Sharman et al. 2008). In recent years there have been important economic losses in sunflower and mungbean crops. However, until recently little was known about the causal strain of TSV or its key alternative hosts in the region. TSV was found to occur commonly in *Parthenium hysterophorus*, as symptomless infections, in central Queensland across a large area infested with this highly invasive and prolific weed (Sharman et al. 2009). Several isolates of TSV collected across the geographic range of *P. hysterophorus* were found to share identical coat protein sequence with each other and with TSV from crop plants in the same area. TSV is a pollen-borne virus and transmission relies on the virus particles from infected pollen entering plant cells through the feeding injury caused by thrips. The central Queensland strain of TSV was shown to be readily transmitted by the commonly collected species, *Frankliniella schultzei* and *Microcephalothrips abdominalis* using TSV-infected *P. hysterophorus* pollen. Seed transmission of TSV in *P. hysterophorus* occurred at rates of 6.8 to 48% and there was almost no change in this rate when *P. hysterophorus* seed was stored for up to 24 ½ months. These results indicate that *P. hysterophorus* is a key alternative host for the development of TSV disease epidemics in surrounding crops in central Queensland.

## References

[bibr03] Sharman M, Thomas JE, Persley DM (2008). First report of *Tobacco streak virus* in sunflower (*Helianthus annuus*), cotton (*Gossypium hirsutum*), chickpea (*Cicer arietinum*) and mung bean (*Vigna radiata*) in Australia.. *Australasian Plant Disease Notes*.

[bibr04] Sharman M, Persley DM, Thomas JE (2009). Distribution in Australia and seed transmission of Tobacco streak virus in Parthenium hysterophorus.. *Plant Disease*.

# Research article: Effect of selected cultural practices in suppressing *Iris yellow spot virus* in onion

**Published:** 2010-10-01

**Authors:** Clinton C. Shock, E.B.G. Feibert, L.D. Saunders, L.B. Jensen, Hanu R. Pappu, S.K. Mohan, R.K. Sampangi

**Affiliations:** ^1^ Oregon State University, Malheur Experiment Station, 595 Onion Ave. Ontario, OR 97914 USA; ^2^ Oregon State University, Malheur County Extension service, 710 SW 5^th^ Ave., Ontario, OR 97914 USA; ^3^ Department of Plant Pathology, Washington State University, 345 Johnson Hall, Pullman, WA 99164-6430 USA; ^4^ University of Idaho, Parma Research and Extension Center, 29603 U of I Fane, Parma, ID 83660 USA

*Iris yellow spot virus* (IYSV) is a major constraint to the production of onion (*Allium cepa*) bulb and seed crops in the Pacific Northwest of the US. First found locally in the Treasure Valley of Idaho and Oregon infecting onion seed crops in 1989, the virus is now reported from many onion-producing areas around the world. Onion plants infected with IYSV can progressively loose leaf area, resulting in reduced yield and reduced bulb size. The virus is transmitted by onion thrips (*Thrips tabaci*). Thrips control has become more difficult because of the increased thrips resistance to insecticides. It is not known whether irrigation and fertilization practices that reduce plant stress might also reduce the impact of IYSV. The combined effects of variety, irrigation system, irrigation criterion, and nitrogen (N) rate on IYSV expression and onion yield and grade were evaluated in 2007 and 2008. Fertilization at 112 kg N/ha resulted in a higher onion yield and grade in 2007 than 224 kg N/ha. There were no differences in onion yield or grade between N rates in 2008, but N failed to influence disease incidence either year. Symptoms of IYSV were fewer in 2007 and no significant differences between treatments were observed. In 2008, averaged over varieties and N rates, drier irrigation criteria (higher SWT) resulted in significantly more severe IYSV symptoms. Averaged over varieties and N rates, drip irrigation at 30 kPa resulted in significantly lower marketable, colossal, and colossal plus super-colossal bulb yield than the wetter irrigation criteria in both 2007 and 2008, but the differences were more pronounced in the presence of IYSV in 2008. There were no significant interactions between variety, irrigation criteria, and N rate either year.

# Research article: Host regulation by *Thripinema fuscum* and effects on *Frankliniella fusca* population dynamics

**Published:** 2010-10-01

**Authors:** Kelly Sims, J. Funderburk, S. Reitz, D. Boucias

**Affiliations:** ^1^ Department of Entomology and Nematology, University of Florida, 970 Natural Area Drive, Gainesville, Florida 32611, USA; ^2^ North Florida Research and Education Center, University of Florida, 155 Research Road, Quincy, Florida 32351, USA; ^3^ USDA-ARS, Center for Biological Control, Florida A&M University, Fallahassee, Florida 32307, USA

The tobacco thrips *Frankliniella fusca* (Hinds) (Thysanoptera: Thripidae) is a polyphagous insect pest of numerous fruit, vegetable, and ornamental crops. Significantly, *F. fusca* is known to cause extensive economic damage in various cropping systems by transmitting *Tomato spotted wilt virus* (Bunyaviridae: Tospovirus). The discovery of the entomogenous nematode *Thripinema fuscum* Tipping & Nguyen (Tylenchida: Allantonematidae) parasitizing *F. fusca* implicated it as a potential biological control agent of viruliferous thrips in agroecosystems. *Thripinema* spp. are specialized obligate parasites that attack their hosts within the moist microhabitats of plant structures. Importantly, *T. fuscum* reduces host feeding, induces female sterility, and reduces the competency of this insect vector to transmit *Tomato spotted wilt virus*. These events all occur with negligible effects on thrips longevity and survival. *Thripinema* are intrinsically capable of suppressing populations of thrips and cause near-extinction of local populations in agroecosystems. The host-parasite biology of *F. fusca* and *T. fuscum*, including the effects of parasitism on host population dynamics, will be discussed. In addition, the potential mechanisms by which these parasites interfere with insect vector populations will be explored. Understanding the biological interactions between hosts and parasites can be used to enhance current understanding of the mechanisms utilised by parasites to succeed in fluctuating host populations and are important determinants for successfully developing ecologically based management programs.

# Research article: Detection of multiple virus infection with severe mosaic affected watermelon from Bikaner (India)

**Published:** 2010-10-01

**Authors:** P.N. Sivalingam, T.A. More, R.K. Jain

**Affiliations:** ^1^ Central Institute for Arid Horticulture, Bikaner — 334 006, India; ^2^ Unit of Plant Virology, Division of Plant Pathology, Indian Agricultural Research Institute, New Delhi — 110 012, India

A disease of watermelon (*Citrullus lanatus* (Thunb.) Matsumura and Nakai) characterised by severe mosaic, leaf reduction and shoe-stringing, upright branches, shortening of internodal length, and bud necrosis was observed during 2008 at the Central Institute for Arid Horticulture experimental farm near Bikaner, India, with disease incidence of more than 80%. Symptomatic watermelon plants were tested for the presence of virus by electron microscopy (EM) and direct antigen coated —ELISA (DAC-ELISA) using polyclonal antiserum to *Tobacco streak virus* (TSV), *Watermelon bud necrosis virus* (WBNV), *Cucumber mosaic virus* (CMV) and *Papaya ringspot virus* (PRSV). Flexuous and isometric particles were seen under EM. In DAC-ELISA, symptomatic watermelon samples reacted strongly with WBNV, CMV and PRSV antisera. Asymptomatic samples of other cucurbitaceous crops such as *Citrullus colocynthis* (Tumba), *Cucumis melo* (kachri) and *Lagenaria siceraria* (bottle gourd) were also showed the presence of flexuous and isometric particles under EM and were ELISA positive for WBNV, CMV and PRSV. The preliminary results suggested co-infection of Tospo-, Cucumo- and Poty-viruses with watermelon and *C. colocynthis, C. melo* and *L. siceraria* may serve as alternative hosts for these viruses.

# Research article: Efficient mechanical transmission of *Iris yellow spot virus* (IYSV) to onion and their detection by tissue blot immunoassay (TBIA)

**Published:** 2010-10-01

**Authors:** Sundaraj Sivamani, S. Krishnaveni, Z.B. Usha, K.S. Ravi

**Affiliations:** ^1^ Department of Plant Molecular Biology and Biotechnology, Tamil Nadu Agricultural University, Coimbatore-641003, Tamil Nadu, India; ^2^ Molecular Virology, Mahyco Research Centre, Dawalwadi, Jalna, Maharashtra-431203, India

IYSV emerged as a major limiting factor in successful cultivation of onion bulb and seed crops in India. Difficulties were encountered to detect IYSV in onion due to uneven distribution. In this study a simple and rapid detection method tissue blot immunoassay (TBIA) was developed using polyclonal antibody prepared against recombinant nucleocapsid protein (NP) of IYSV. The TBIA result was comparable to the DAS-ELISA for detection of IYSV in onion bulb and seed crops. Use of resistant cultivar is the most effective strategy for the management of tospoviruses. Cultivar screening and breeding trials were found to be slowed down in onion due to the difficulties in production of disease symptoms by IYSV. Successful inoculation of IYSV to individual plants by mechanical methods is necessary to evaluate the level of resistance in a genotype to be used in breeding programs. In the present study, an improved mechanical transmission protocol was developed by using efficient source of inoculum, antioxidant, abrasives and growth stages. Identification of critical parameters resulted in successful production of disease symptoms observed under field conditions in onion and consistently higher rate of transmission (93.3%) was achieved. Further the mechanical transmission of IYSV to commercially available onion varieties resulted in selection of cultivars based on symptom severity. The detection method TBIA and mechanical inoculation procedure developed in the present study facilitates to understand disease epidemiology and quick identification of resistance genotypes under controlled conditions.

# Research article: Utilising *Lisianthus* (*Eustoma grandiflorum*) as an indicator host model system to evaluate *Iris yellow spot virus* and its interactions with *Thrips tabaci*

**Published:** 2010-10-01

**Authors:** Rajagopalbab Srinivasan, S. Diffie, S. Mullis, D.G. Riley, R.D. Gitaitis, H.R. Pappu

**Affiliations:** ^1^ Department of Entomology, University of Georgia, Tifton, GA 31794; ^2^ Department of Plant Pathology, University of Georgia, Tifton, GA 31794; ^3^ Department of Plant Pathology, Washington State University, Pullman, WA 99164, USA

*Iris yellow spot virus* (IYSV) (family *Bunyaviridae*; genus *Tospovirus*) is a serious pathogen threatening onion production in the United States. Information on IYSV-thrips-host interactions is lacking. Onion has been a less than ideal host for studying the biology of IYSV as it is relatively difficult to grow in a greenhouse, does not always produce symptoms upon infection by IYSV, and it is not easy to mechanically inoculate with the virus. In this study we used *Lisianthus* (*Eustoma grandiflorum*), as an indicator host and address issues such as transmission by nymphs and adults of *Thrips tabaci*, mechanical transmission, virus distribution, and the effect of temperature on IYSV symptom expression. To optimise a transmission protocol using *T. tabaci* nymphs and adults, experiments were conducted using Mylar film cages (one cage /*Lisianthus* plant) with a copper mesh top (178 microns). Ten adults or nymphs were used to inoculate each plant. IYSV-infected nymphs and adults were collected from IYSV-positive onion plants and non-infected thrips from healthy onion plants. *Thrips tabaci* adults more efficiently transmitted IYSV (75%) than nymphs (56.6%). Mechanical inoculations were conducted on *Lisianthus* from IYSV-infected onions and *Lisianthus. Lisianthus* to *Lisianthus* inoculation resulted in higher transmission (80%) than from onion to *Lisianthus* (68%). In both cases, typical sunken round chlorotic spots appeared 3–5 days post inoculation, and then progressed into a systemic infection. To assess the virus distribution, samples were taken from leaves, stem, and roots and tested for IYSV by ELISA. All the plant parts tested were positive for the virus. The effect of three different temperature regimes 18–23°C, 25–30°C, and 30–37°C, with a 12h day-length, were tested for IYSV infection and symptom expression. The optimum temperature range for was found to be 25–30°C.

# Research article: Attractiveness of adult *Thrips tabaci* to IYSV infected plant

**Published:** 2010-10-01

**Authors:** Jun Sofue, Tamotsu Murai

**Affiliations:** Faculty of Agriculture, Utsunomiya University, 350 Mine, Utsunomiya, Tochigi, Japan 321-8505

It is well known that *Tospoviruses* are specifically transmitted by some thrips species (*Thripidae*). This specific interaction between virus and thrips suggests a hypothesis that the viruses induce attractive behaviour of thrips to infected plants. This study begins to verify whether this hypothesis is correct or not. In fact, it is known that more thrips are observed on the virus infested leaves than intact leaves. In a choice test that used *Tomato spotted wilt virus* (TSWV) and *Frankliniella occidentalis*, more thrips were observed on infected plant(pepper) (Maris et al., 2004). We also observed the attractiveness of *Thrips tabaci* to the plant infected by *Iris yellow spot virus* (IYSV), which is transmitted by *T. tabaci* specifically. The attractiveness of *T. tabaci* and *F. occidentalis* to IYSV infected plant was tested in a dark condition. *Impatiens walleriana* was used as a test plant, which has local legion of IYSV transmitted by *T. tabaci* nor *F. occidentalis. T. tabaci* was significantly attracted to IYSV infected leaves, however *F. occidentalis* was not. The result suggests that there is a tritrofic interaction among IYSV, *T. tabaci* and infected plant. Thus, virus attracted the thrips that have an ability of transmit them more efficiently.

# Research article: Thrips species composition shift in the Vidalia onion production region of Georgia, USA

**Published:** 2010-10-01

**Authors:** Alton Sparks, S. Diffie, D. Riley

**Affiliations:** University of Georgia, Tifton Campus, Tifton, GA, USA, 30602

Thrips are the primary arthropod pest of onions in Georgia, USA. Historically the thrips complex on onions in Georgia has been reported to consist of three species: onion thrips, *Thrips tabaci*; tobacco thrips, *Frankliniella fusca*; and the western flower thrips, *F. occidentalis*. Onion thrips has historically been of little consequence in this region, as the thrips populations on onions typically consist of less than 1 percent onion thrips. In the fall of 2003, *Iris yellow spot virus* (IYSV) was detected in the Vidalia onion growing region. This area produces the vast majority of onions in Georgia, with an annual farm gate value of approximately 100 million dollars. Since the onion thrips is the primary vector of IYSV, populations of this species received new interest. In addition, onion thrips were detected in culls from onions imported into Georgia from Peru. The potential for establishment and spread of a new biotype of onion thrips also fueled interest in monitoring of thrips on onions within this region. Thrips species composition has been monitored within the Vidalia production region for the past six years. A marked shift in species composition has been documented in the last three years. The tobacco thrips represented over 95 percent of thrips collected from commercial onion fields in the first three years of this study. The onion thrips increased dramatically in the last three years, with individual fields in which onion thrips were the predominant species. Potential reasons for this species shift include introduction of a new biotype of onion thrips and insecticide selection.

# Research article: Chemical and cultural management practices for thrips and *Tomato spotted wilt virus* in Georgia, USA

**Published:** 2010-10-01

**Authors:** Alton Sparks, D. Riley, D. Langston

**Affiliations:** University of Georgia, Tifton Campus, Tifton, GA, USA, 30602

Thrips-vectored *Tomato spotted wilt virus* (TSWV) has become one of the major causes of diseases of crops in the southeastern United States, causing as much as $100 million in damage in a single year. TSWV is transmitted in the southeastern U.S. primarily by tobacco thrips, *Frankliniella fusca*, and western flower thrips, *Frankliniella occidentalis*. Among vegetable crops in this region, fresh market tomatoes and peppers are at greatest risk. Although multiple tactics are available for management of this problem, growers currently rely heavily on resistant varieties, which contain a single source of resistance. If this resistance fails, growers will be forced to rely on chemical and cultural management. Recommendations for management of TSWV in tomatoes include use of UV-reflective mulch, imidacloprid applied at transplanting, and foliar applications of acibenzolar-S-methyl. Each of these tools has potential costs and benefits associated with their implementation. Current research being conducted under a USDA-RAMP project includes evaluation of these management tools in small plot replicated trials and large plot demonstration plots in south Georgia. These trials include comparisons of treatment combinations of three plastic mulches (black, UV-reflective, and heatstripe), imidacloprid at transplanting (versus no systemic insecticide), and pre-bloom applications of acibenzolar-S-methyl (with multiple timing of applications in the small plot trials, versus non-treated in all trials). The effects of these management practices on densities of thrips, TSWV infection, and yield are being evaluated and will be discussed.

# Research article: Tospoviruses in New Zealand

**Published:** 2010-10-01

**Authors:** Joe Tang, G.R.G. Clover

**Affiliations:** Plant Health and Environment Laboratory, MAF Biosecurity New Zealand PO Box 2095, Auckland 1140, New Zealand

Three species of *Tospovirus* have been recorded in New Zealand: *Impatiens necrotic spot virus* (INSV), *Iris yellow spot virus* (IYSV) and *Tomato spotted wilt virus* (TSWV). TSWV occurs throughout New Zealand where it has caused serious sporadic epidemics in vegetable crops such as tomato, capsicum and potato since it was first detected in 1936. In contrast, the incursions of IYSV and INSV were reported recently, in 2007 and 2003, respectively. IYSV has been found widespread in onion and shallot crops through the North and South Islands during a survey in 2008. At least 10 species of glasshouse ornamentals have been found to be infected with LNSV since 2003, including begonia, cyclamen, freesia, gardenia, gerbera, hibiscus, hoya, impatiens, primula and ranunculus. The distribution and activity of two principal vectors, onion thrips (*Thrips tabaci*) and western flower thrips (*Frankliniella occidentalis*), are thought to be linked to outbreaks of the three tospoviruses.

# Research article: Latest lucid identification tools

**Published:** 2010-10-01

**Authors:** Matt Taylor

**Affiliations:** Centre for Biological Information Technology (CBIT), The University of Queensland, Australia

This year marks the 10^th^ anniversary of Lucid — a digital, matrix key system developed at The University of Queensland. Over this period, Lucid has evolved to keep pace with technological advances in software. Version 3, in particular, marked a significant turning point with a complete change in technology; Lucid was rewritten under the Java platform and released with a host of new features (E.g. feature and entity hierarchies, scoping and positive dependencies). Lucid 3.5, the next major release, substantially advances the Lucid platform, including key reporting, key and player customisations, media importing, and the creation of natural language descriptions from the matrix data. Focusing on one of these major new features, natural language creation (where descriptive text is derived from matrix scores), our goals were threefold: 1) make the process as easy as possible, 2) use open standards, 3) be as flexible as possible. The success of Lucid natural language creation lies in its template builder, which enables authors to quickly and easily structure and build a description, using widely available and understood XML technology and standards. In addition, several example formatting templates can be used ‘off-the-shelf’ or be easily customised. Descriptions can be rapidly created for publication or generated as interactive descriptive fact sheets that include multimedia, which if desired, can automatically be attached to the key. The list of new features and improvements under development is extensive, with many ideas and suggestions flowing on from our users. Improved image display options and specimen data handling are two such features planned for future updates. With recent improvements in Internet speed, Connectivity and computing power, along with the desire for global collaborative key building and data aggregation, the need for a platform that fulfils this seems clear. To this end CBIT is working with the Encyclopaedia of Life and the Atlas of Living Australia on a project called IdentifyLife. One goal of this project is to develop an identification system capable of scaling to all living organisms, while not to forgetting the end-user of this information — the person asking ‘What is this?’

# Research article: Too cold to smell? Temperature-dependent responses by *Cycadothrips* to cycad cone host cues

**Published:** 2010-10-01

**Authors:** Irene Terry, G. Walter, C. Moore, R. Roemer

**Affiliations:** ^1^ University of Utah, Department of Biology, Salt Fake City, UT 824112, USA; ^2^ University of Queensland School of Biological Sciences, Brisbane, St. Lucia 4072, AUS; ^3^ University of Utah, Department of Mechanical Engineering, Salt Lake City, UT 84112 USA

*Cycadothrips* sp. (Aeolothripidae) are the sole pollinators of several *Macrozamia* cycad species and they are dependent upon male cones that provide both food (pollen) and nursery facilities to complete larval development. Cones have a diel thermogenic peak which coincides with peak volatile emissions and the rapid flight of thrips from cones. Later in the day, pollen-bearing thrips return to a cycad cone, sometimes a female cone. We have been investigating what motivates thrips to leave their ‘home’ and food. Experiments to determine the effects of cone temperature alone on thrips behaviour, by heating a thrips—bearing cone exogenously, were unsuccessful because increasing temperatures around cones induces thermogenesis which increases volatiles. Thrips did not respond to odours applied to cold cones, enclosed in glass. Thus, we resorted to Y-tube olfactometry experiments. Initial experiments showed that at 22°C, light attracts thrips, and β-myrcene slightly attracts at low concentrations and repels at high concentrations. However, the effects of β-myrcene and light are temperature-dependent and several factors must cooccur to induce thrips to leave cones. At warm ambient temperatures (≥∼22°C) thrips are active and are attracted to light, and their response to β-myrcene is concentration dependent (previously described). At cold temperatures (≤18°C), thrips avoid light and do not respond to β-myrcene regardless of the concentration (within ecologically relevant levels). This mirrors field observations of thrips and cones. When it is cold during the day in habitat (<∼20°C), then cones do not become thermogenic, and thrips do not leave cones. When thrips are shaken from cold cones, they move away from light and toward cones. At warmer ambient temperatures (≥∼22°C), cones become thermogenic, volatile emissions increase, and thrips move to the surface of the cone, become very active for a few minutes and then take flight.

# Research article: Made for one another: *Cycadothrips*, pollen and Australian *Macrozamia* cycads

**Published:** 2010-10-01

**Authors:** Irene Terry, G. Walter, C. Moore, R. Roemer

**Affiliations:** ^1^ University of Utah, Department of Biology, Salt Fake City, UT 824112, USA; ^2^ University of Queensland School of Biological Sciences, Brisbane, St. Lucia 4072, AUS; ^3^ University of Utah, Department of Mechanical Engineering, Salt Fake City, UT 84112 USA

*Cycadothrips* sp. (Aeolothripidae) and some species of the cycad genus, *Macrozamia* (Cycadales: Zamiaceae), all Australian endemics, are dependent mutualists. In these systems, only male cones provide food (pollen) and shelter for adults and larvae, and adult thrips vector pollen to female cones. These plant—insect interactions suggest a coevolved system, with specific plant traits that affect thrips behaviours. Cones have a diel thermogenic episode that occurs during the middle of the day, cones emit strong odours (mainly β-myrcene) during thermogenesis, and thrips take flight from the cones in large numbers. Later during the day, thrips return to cones, sometimes to a female cone where thrips deposit pollen on the ovules. Male and female cones have a similar timing of cone thermogenesis and similar odour components, so females with no known reward may dupe the insects to visit and leave pollen. These specific traits and insect behaviours contrast with some *Macrozamia* species that are pollinated only by *Tranes* sp. weevils. In these systems, the cones are thermogenic during the early evening, coinciding with increased volatile emissions (different chemistry from thripspollinated cones) and weevil flight away from the cones. Pollination by thrips appears to be very efficient, with relatively few thrips required per cone to pollinate nearly all eggs in the cone. In addition, because thrips feed on pollen only they don't damage any other cone tissues. *Tranes* can damage female tissue because they sometimes feed on sporophylls and ovules. In this talk, we will present some of our pollination methods and results, discuss the implications of our results in terms of efficiency of the pollination system, summarise what we know about *Cycadothrips* and their closest relatives, and review what we know about pollination of plants by thrips.

# Research article: The lure of scent: allelochemicals for thrips pest management

**Published:** 2010-10-01

**Authors:** David A.J. Teulon, M.M. Davidson, M-C. Nielsen, N.B. Perry, R.W.H.M. van Tol, W.J. de Kogel

**Affiliations:** ^1^ New Zealand Institute for Plant & Food Research Limited, Private Bag 4704, Christchurch, New Zealand; ^2^ Plant Research International, P.O. Box 69, 6700 AB, Wageningen, the Netherlands

There is considerable potential to utilise semiochemicals (pheromones and alleochemicals: allomones; kairomones; synomones) to develop new tools for thrips pest management such as improved monitoring (including surveillance for invasive species), push-pull, lure and kill, lure and infect and other technologies. This paper focuses on allelochemicals as thrips attractants. It reviews recent research on the intrinsic (e.g. host finding behaviour, species specific responses, physiology) and extrinsic (e.g. temperature, dose, mixtures, competing host plant odours) factors influencing thrips responses to allelochemical lures. It places this current knowledge in a historical context with a view to maximising the use of allelochemicals for thrips pest management.

# Research article: Diversity and abundance of thrips (Thysanoptera) associated with leaf-litter and bark across forest and tree types in southeast Queensland

**Published:** 2010-10-01

**Authors:** Desley J. Free

**Affiliations:** Department of Employment, Economic Development and Innovation, Entomology Collection, 80 Meiers Rd, Indooroopilly, Qld 4068.

Thrips (Thysanoptera) associated with leaf-litter in Brisbane Forest Park, southeast Queensland, are demonstrated to occur in higher numbers and greater diversity in dry sclerophyll forests than in wet sclerophyll forest or rainforest. Further sampling in dry sclerophyll forests screened for thrips associated with leaf-litter under two dominant tree species, *Eucalyptus major* and *Acacia melanoxylon*, and simultaneously for thrips associated with the bark of each. *Eucalyptus major* hosted greater numbers of thrips individuals and *Acacia melanoxylon* a greater diversity of thrips species associated with their leaf-litter and bark. A similar sampling program across two common eucalypts with different bark types, *Eucalyptus major* — grey gum with flaky bark and *Eucalyptus siderophloia* — grey ironbark with rough bark, revealed little difference between the numbers of thrips individuals and species associated with each. Thrips species associated with leaf-litter are believed to feed on fungi, however little is known of the biology of thrips associated with bark of living trees. This study demonstrates a great diversity of dry sclerophyll fungivores in an insect order that contains only one major family of fungal-feeders, Phlaeothripidae, the most species rich family in Thysanoptera.

# Research article: *Dictyothrips betae*, a new tospovirus transmitting thrips species

**Published:** 2010-10-01

**Authors:** Massimo Turnia, M. Ciuffo, F. Bosco, G. Mautino, L. Tavella

**Affiliations:** ^1^ Istituto di Virologia Vegetale, Sez. di Torino, CNR, Strada delle Cacce n73, Torino 10135, Italy; ^2^ Di.Va.P.R.A. Entomologia e Zoologia applicate all'Ambiente, University of Torino — Via F. da Vinci 44, 10095 Grugliasco (TO), Italy

*Dictyothrips betae* is the predominant thrips species on *Polygonum convolvulus* and *P. dumetorum* plants infected with a recently characterised tospovirus species, *Polygonum ringspot virus* (PolRSV). Transmission experiments in controlled environment (leaf disk assays) with adults collected directly in the field demonstrated the competence of this thrips to transmit PolRSV even if with only a 4% transmission rate. However, this percentage increased to 16% using newly hatched larvae fed on infected leaves for 3 days, and reared on leaf disks substituted and assayed by DAS-ELISA every 3 days. During transmission experiments, insect life cycle was also studied. By contrast, *Frankliniella occidentalis* and *Thrips tabaci* failed to transmit PolRSV in leaf disk assays. RT-PCR with specific primers for the nucleocapsid (N) protein and western blot analysis of adult thrips to detect the N and NSs proteins confirmed the presence of the virus in *D. betae* individuals after feeding for at least 5 days on healthy plants. Partial sequences of mitochondrial COI, nuclear 28S ribosomal DNA and the elongation factor-1a were used for phylogenetic analysis of *D. betae*. The results confirmed a phylogenetic relationship between this species and tospovirus-transmitting insects of the genus *Thrips*. The uniqueness of the strict ecological relationship between PolRSV, its vector *D. betae*, and its plant host is underlined.

# Research article: Thrips vectors and tospoviruses: the rendezvous with destiny

**Published:** 2010-10-01

**Authors:** Diane Ullman

**Affiliations:** Department of Entomology and Office of the Dean, College of Agricultural and Environmental Sciences, University of California, One Shields Avenue, Davis, California 95616, USA

Consider the chance meeting of two entities: the thrips (Thysanoptera: Thripidae), members of the Insecta, undoubtedly the most abundant and diverse group of animals on the planet; and the progenitor of the tospoviruses (Bunyaviridae: *Tospovirus*): simple, elegant, membrane-bound packages of protein and RNA, the planet's experiment in genomic strategies. The intimate relationship co-evolving between these biological entities brings us at least 15 tospovirus species transmitted by at least 10 species of thrips to food, fiber and ornamental crops worldwide. In their rendezvous with destiny, new thrips vectors and tospoviruses continue to arise, leaving a path of destruction around the globe. The need to protect crops against this ever shifting and recalcitrant threat first brought scientists together in a USDA Workshop in 1987, and draws us to Australia for *The IX International Symposium on Thysanoptera and Tospoviruses*. In the past 20 years, a long path of discoveries has been built: from a single virus *Tomato spotted wilt virus* (TSWV) in a monotypic family to a wide array of diverse plant viruses placed taxonomically within the Bunyaviridae, a family of animal viruses; from a handful of thrips vectors to 10 thrips species with highly specific, propagative vector/virus relationships; and from thrips simply transporting a virus dose to an intimate, intricate co-evolving virus/host relationship. Current knowledge revealing the interplay between thrips, tospoviruses and their shared plant hosts will be highlighted in the context of vector biology, functional genomics of tospoviruses, thrips transcriptomics and development of control strategies for thrips-transmitted tospoviruses. In spite of advances in our understanding of thrips-virus-plant interactions, our global community of entomologists, plant pathologists and crop specialists has yet to create significant solutions for direct damage done by thrips or disease problems caused by their intimate relationship with tospoviruses. As we work together at this conference and in days and years to follow, I challenge each of us to reach for innovation, to reach for the sharing of knowledge and the creative application of scientific advances to strategies to control thrips and tospoviruses.

# Research article: Human antibody genes confer resistance to tospovirus (PBNV) in tomato

**Published:** 2010-10-01

**Authors:** Akella Vani, K. Jagadish, B.J. Nirmal Kumar

**Affiliations:** Department of Biotechnology, Indian Institute of Horticultural Research, Hessarghatta Fake post, Bangalore-560089, India

*Peanut bud necrosis virus* (PBNV) is one of the economically important tospoviruses infecting tomato in India. PBNV causes yield losses up to 80% in tomato. The monoclonal antibodies were generated against the nucleocapsid protein of PBNV using human synthetic single chain antibody library (MRC, London). The antibody genes were isolated and cloned into plant expression construct with plant promoter and transcription terminator. Transgenic tomatoes were generated and evaluated for the presence of the gene, its expression and level of resistance. Plants that were positive for the presence of the transgene but with no detectable level of the protein in an ELISA were susceptible, while plants which had detectable levels were resistant to the virus when inoculated by sap from infected tissue.

# Research article: Studies on thrips of grapes in South India, with special Reference to Scirtothrips dorsalis Hood

**Published:** 2010-10-01

**Authors:** Abraham Verghese, R. Harish

**Affiliations:** Division of Entomology, Indian Institute of Horticultural Research, Bangalore-560089, India

Grape is an important fruit crop in India. In the 1980s, surveys revealed that *Rhiphorothrips cruentatis* Hood was the main species infesting the leaves and fruits of grapes. However, by the late 1990s, *R. cruentatis* was completely replaced by *Scirtothrips dorsalis* Hood, a probable ecological homologue. *Scirtothrips dorsalis* causes serious ‘scab’ symptoms on fruits resulting in 20–30% yield loss and reduction in marketability. Since 2001, a systematic programme has been conducted, with the objectives to study the thrips species complex, bioecology and seasonal incidence of *Scirtothrips dorsalis* in relation to climate, extent of damage and develop suitable management. Surveys in south India recorded five species of thrips in grape ecosystem belonging to three different families; *Scirtothrips dorsalis* Hood, *Streothrips arorai* Bhatti, *Haplothrips tenuipennis* Bagnall, *Karnyothrips flavipes* Jones and *Xylaplothrips* sp. The last four are new records for India (identified by L, A. Mound, Australia). The phenology of the crop played a significant role in determining the level of thrips infestation. The density of *S. dorsalis* and the four other species together reached a peak [12.92/shoot (*S. dorsalis*) and 0.74/shoot (other thrips put together) during winter] at 30–40 days after pruning when the crop was in tender leaf stage and flowering had initiated. In summer also peak populations of *S. dorsalis* (16.48/shoot) and other four species of thrips together (0.80/shoot) coincided with tender leaf and flowering stages. Thus tender leaves were significantly and positively correlated to thrips density, while fully matured leaves, small, medium and large-sized fruits had negative correlation. This information was crucial in timing spray interventions at tender leaf stage. *S. dorsalis* took 14.35 ± 3.18 days to complete a life cycle. Thus, the first 2–3 generations are important in grapes. *S. dorsalis* infestation was positively influenced by temperature (both maximum and minimum). Relative humidity, rainfall and wind speed had negative effect. Higher temperatures therefore, increase the population. This is a possible reason for *S. dorsalis* to have displaced *R. cruentatis*, as summer temperatures have marginally risen in several grape growing areas. In summer, *S. dorsalis* is serious; the damage on *cv* Bangalore Blue (a table and juice variety) was 5.67 % and 33.18 % in winter and summer, respectively. The efficacy of synthetic, contact and botanical insecticides *viz*., acephate 75%SP, verticel 100 SP, endosulfan 35 EC, chlorpyriphos 20 EC, carbaryl 50 % WDP, cartap hydrochloride 5 % SP, frpronil 5 % SC, fish oil rosin soap, azadirachtin 0.03% EC, and endosulafan + azadirachtin were evaluated against *S. dorsalis* on grapes using two foliar applications at 10 day intervals for two years. Endosulfan followed by cartap hydrochloride and carbaryl effectively controlled *S. dorsalis* during both seasons, whereas, frpronil, endosulfan + azadirachtin and acephate had moderate effect and verticel was least effective. Cartap hydrochloride being more eco-friendly is recommended for thrips management. Based on these findings, the management strategies will be discussed, including aspects on future thrusts like yellow sticky traps, biological control and alternative hosts.

# Research article: *Iris yellow spot virus* on onions in the Murrumbidgee Irrigation Area of New South Wales

**Published:** 2010-10-01

**Authors:** Andrew Watson, J. Mo

**Affiliations:** Yanco Agricultural Institute, Yanco, New South Wales 2705, Australia

Onion (*Allium cepa* L.) production is an important industry in the Murrumbidgee Irrigation Area (MIA) of New South Wales. The region is characterised by a Mediterranean style climate with hot summers and cool winters. Mean rainfall is close to 30mm per month and therefore crops require irrigation. Onions are grown for the fresh market with planting taking place in May to June with the early harvest being carried out in late November to December and the late harvest in February/March. The region also has a thriving vegetable seed industry where onion seed is also produced. *Iris yellow spot virus* (IYSV) is a tospovirus that is transmitted by onion thrips and affects onions, leeks, iris and lisianthus. The disease was found in the United States in 1989 and has since been found in Australia, New Zealand and many other countries. IYSV does not cause death of plants but instead weakens plants and makes them more susceptible to nutritional and water stress.

IYSV was reported from three states in Australia for the first time in 2002. One of these reports was from an onion seed crop in the MIA that exhibited typical IYSV lesions on the flower stalks. Since 2002 the disease has not been implicated in yield loss in onions. In 2009 a commercial organic onion crop in the MIA showed typical symptoms of IYSV. Samples were taken and the virus was positively identified by ELISA. The onions were a late crop and a combination of moisture and nutritional stress together with IYSV affected the crop severely. This is the first report of an onion crop seriously affected by IYSV in Australia.

# Research article: *Tomato spotted wilt virus* (TSWV) vector thrips preference and oviposition performance in potato

**Published:** 2010-10-01

**Authors:** G. Westmore, G.R. Allen, N.W. Davies, Calum R. Wilson

**Affiliations:** ^1^ Tasmanian Institute of Agricultural Research, University of Tasmania, New Town Research Faboratories, 13 St Johns Avenue, New Town, Tasmania 7008; Australia; ^2^ School of Agricultural Science, University of Tasmania, Private Bag 54, Hobart, Tasmania 7001, Australia; ^3^ Central Sciences Faboratory, University of Tasmania, Private Bag 74, Hobart, Tasmania 7001, Australia

Breeding to improve management of virus diseases generally targets the pathogen. However, vector resistance may offer valuable disease control and examples of successfully deployment of vector resistance are described (Jones, 1987). Host preference by thrips is known (Herrin and Warnock, 2002) but the mechanisms are still largely unclear. Visual cues and plant chemistry arguably play major roles in thrips choice (Terry, 1997). Here we determined host preference and reproductive performance of TSWV thrips vectors for potato. We demonstrated differences in cultivar preference by onion thrips (*Thrips tabaci*). Further testing of onion thrips (along with tomato (*Frankliniella schultzei*), and western flower thrips (*F. occidentalis*)) using colour cards, showed distinct colour preferences. Tomato and western flower thrips preferred yellow to all other colours (green, blue, red, white), whilst onion thrips preferred green over yellow, which was preferred to all others. Onion thrips also had preference for light green over mid or dark green. These shades were representative of potato cultivars and reflected cultivar choices.

However, onion thrips also preferred green colour cards to potato leaves (of similar shades), and in the absence of visual cues, retained preference for certain cultivars. These data suggested a role for leaf volatiles (perhaps inhibitory) in thrips choice. Preliminary analyses have suggested differences exist in volatile profiles of the test cultivars, including terpenes previously known to influence insect host location. The relative preference for potato to other plant species was also tested with certain alternative species attracting greater thrips than potato. This result suggests careful selection of alternative host species may facilitate a successful decoy crop system. Distinct differences in onion thrips reproductive performance on potato cultivars were also demonstrated. As has been observed in other insect:host systems, cultivar preference and reproductive performance of onion thrips appeared unlinked.

## References

[bibr05] Jones AT (1987). *Annals of Applied Biology*.

[bibr06] Herrin B, Warnock DF (2002). *HortScience*.

[bibr07] Terry LI (1997). *Host selection, communication and reproductive behaviour*..

# Research article: Genetic variability within Australian onion thrips (*Thrips tabaci* Lindeman) populations and association with TSWV vector capacity

**Published:** 2010-10-01

**Authors:** G. Westmore, F.S. Poke, G.R. Allen, Calum R. Wilson

**Affiliations:** ^1^ Tasmanian Institute of Agricultural Research, University of Tasmania, New Town Research Faboratories, 13 St Johns Avenue, New Town, Tasmania 7008, Australia; ^2^ School of Agricultural Science, University of Tasmania, Private Bag 54, Hobart, Tasmania 7001, Australia

In recent literature doubts over the capacity of onion thrips (*Thrips tabaci* Lindeman) to vector modern strains of TSWV have been raised (eg Whitfield et al., 2005), yet onion thrips remains the only recorded vector species associated with TSWV epidemics in potato in Tasmania, Australia (Wilson 2001). Data from Europe reaffirmed this species as an important vector. However, highly variable transmission efficiencies have been shown with studies suggesting an influence of host-plant preference (Brunner et al., 2004) and the presence of males in the population with thelytokous female-only populations having low or no vector competence (Wijkamp et al., 1995; Chatzivassiliou et al., 2002). Only thelytokous populations of onion thrips have been found in Australia. We examined the ability of populations of onion thrips collected from various host species across Australia to vector TSWV. It was found that only onion thrips populations collected from potato or a capeweed plant within a potato field were capable of vectoring TSWV, with no transmission by populations collected from onion. Molecular variability (mitochondrial COI sequences) within the onion thrips populations was assessed. All vector competent populations (from potato or capeweed) co-located within the same phylogenetic clade, while the populations sourced from onion crops segregated to a different clade. Populations from other hosts not tested for vector capacity (eg Chrysanthemum) were located separately again. These results link differentiation of (preferred) source host, and vector competence to genetic diversity, and suggest geographic distribution is a less important determinant in Australian onion thrips population diversity.

## References

[bibr08] Brunner PC, Chatzivassiliou EK, Katis NI, Frey JE (2004). *Heredity*.

[bibr09] Chatzivassiliou EK, Peters D, Katis NI (2002). *Phytopathology*.

[bibr10] Wijkamp I, Almarza R, Goldbach R, Peters D (1995). *Phytopathology*.

[bibr11] Wilson CR (2001). Plant Pathol..

[bibr12] Whitfield AE, Ullman DE, German TL (2005). *Annual Reviews of Phytopathology*.

# Research article: Plant genotype effects on a host specific thrips and the impact on biological control

**Published:** 2010-10-01

**Authors:** Greg S. Wheeler, D.A. Williams, F.A. Mound

**Affiliations:** ^1^ USDA/ARS/TPRP, 3225 College Ave, Ft Lauderdale, FF, USA 33314; ^2^ Dept of Biology, Texas Christian University, 2300 S. University Dr, Ft Worth, TX, USA 76129; ^3^ CSIRO Entomology PO Box 1700, Canberra, ACT 2601, Australia

A promising thrips, *Pseudophilothrips ichini* (Phlaeothripidae) has been considered for biological control of the invasive weed Brazilian pepper *Schinus terebinthifolius*. This thrips was originally collected from a southern region of Brazil where it was frequently found associated with significant damage to its host. Subsequently this thrips was introduced into quarantine in Florida, USA to determine its safety for field release to control this weed. However, a major limitation of the thrips population tested in quarantine was its apparent nutritional incompatibility with the genetic varieties of the host that occur in Florida. Although this thrips was collected on the host in Brazil, its survival was less than 5% when fed the Florida variety of Brazilian pepper. Extensive DNA and morphological analysis of the thrips has determined that the quarantined species is incorrectly identified and constitutes a new cryptic species *Pseudophilothrips* n. sp. Chloroplast DNA analysis of the host revealed 14 genetic varieties and the discovery that the new species of thrips was both limited geographically in Brazil and nutritionally to two Brazilian host varieties, neither of which occur in Florida. As a result of these studies, individuals of the species *P. ichini* have been correctly identified in Brazil. These thrips were found feeding on the Florida variety of the host in Brazil. Populations of *P. ichini* have been colonised and are undergoing quarantine testing in Florida to determine suitability for release to control the weed. By revealing thrips x host plant genetic compatibilities, these results have directed the next phase of the quarantine testing of this invasive weed that show promise for controlling this invasive weed in Florida.

# Research article: *Tomato spotted wilt virus* (TSWV) in potato: epidemiology and host resistance

**Published:** 2010-10-01

**Authors:** Calum R. Wilson, A.J. Wilson, F.S. Poke, G. Westmore

**Affiliations:** Tasmanian Institute of Agricultural Research, University of Tasmania, New Town Research Faboratories, 13 St Johns Avenue, New Town, Tasmania 7008, Australia

TSWV Infection in potato is sporadic, but losses are considerable when epidemics occur due to disease severity. Foliar symptoms include leaf necrotic spotting, while infected tubers may show brown flecking to widespread necrosis. TSWV infections can kill young plants. Only two vector species, onion thrips (*Thrips tabaci*) and tomato thrips (*Frankliniella schultzei*), are routinely associated with TSWV epidemics in Australian potatoes (Wilson, 2001). However, population density of migrating thrips is a poor indicator of virus threat. TSWV sources driving epidemics in potato initiate from outside the crop. Seed tubers from infected crops may carry TSWV and replanting can lead to significant virus incidence from tuber-borne sources. However, inefficient virus translocation to and from tubers and self-elimination of infected plants results in removal of most seed-borne inoculum by the following season. Distinct spatial patterns of TSWV infection observed in potato associate with region. An edge effect is obvious in some regions whereby the outermost rows succumb to infection suggesting movement of inoculum from relatively close proximity. Alternately, in other regions epidemics are characterised by random distribution of infections throughout the crop suggestive of inoculum arriving from greater distance. With few exceptions there is little evidence for secondary infections within potato crops despite experimental data showing capacity for spread to and from potato. Variation in virus infection rate has been observed between cultivars but is unlikely to provide significant control. In contrast substantial differences in the efficiency of TSWV translocation from infected foliage to tubers and from infected tubers to progeny plants has been demonstrated (Wilson, 2001). Translocation of TSWV was also influenced by plant age at infection and by environmental conditions. Such resistance to long distance transport of viral pathogens takes on greater importance in a vegetatively propagated root crop like potato.

## References

[bibr13] Wilson CR (2001). *Plant Pathology*.

# Research article: Molecular characters of nuclear ITS2 and mitochondrial COI in thrips, and the application to identify thrips in imported agricultural products

**Published:** 2010-10-01

**Authors:** Wen-Bin Yeh, Mei-Zung Tseng, Ym-Yin Lial, Niann-Tai Chan

**Affiliations:** ^1^ Department of Entomology, 250, Kuo Kuang Rd., Taichung 402, Taiwan; ^2^ Department of Plant medicine, National Pingtung University of Science and Technology; No .1, Hseah-Fu Rd., Neipu, Pingtung, Taiwan 912, R.O.C.

Molecular characters of nuclear intergenic spacer 2 (ITS2) and mitochondrial cytochrome oxidase I (COI) in thrips were established and applied to identify the unknown thrips commonly found in crops. Thrips samples were collected from localities in Taiwan, China, Thailand Vietnam, Japan, and American. Nucleotide sequences of ITS2 from ∼500 individuals within 60 economically important thrips were established. Sequence variations among individuals within species were up to 5%, and that between species within a given genus were more than 6%, and that among genera were up to 60%. Moreover, sequence divergences of COI region of ∼50 species among 160 individuals were less than 2% within species, and that between species is mostly larger than 6%. The recognisable sequence variation between species of the above two amplicons showed that molecular characters were possibly utilised in thrips identification. The 96 microplate and full-automatic capillary electrophoresis were carried out for DNA extraction and PCR product examination of ∼800 thrips individuals concealed in the imported agricultural products. Two specific paired primers were used to examine *Frankliniella occidentalis* and *Thrips tabaci*, which were the most commonly found thrips in the imported agricultural products, but the former had the necessary quarantine. The results showed 125 *F. occidentalis* and 333 *T. tabaci*, but 33 samples revealed the amplified products of both thrips. However, PCR products could not be obtained in 277 samples, perhaps due to non-optimal specimen preservation or different thrips species. Molecular identification results shown, it should be important to include the positive amplified marker simultaneously when thrips samples were not belonging to the examined thrips.

# Research article: Phytogeny of Thripinae using 28S and 18S ribosomal DNA sequences

**Published:** 2010-10-01

**Authors:** Wen-Bin Yeh, Yi-Sheng Tsai, Mei-Zung Tseng

**Affiliations:** Department of Entomology, 250, Kuo Kuang Rd., Taichung 402, Taiwan

Thrips are very important agricultural pests. However, the phylogenetic relationship in thrips has been discussed rarely. In this study, we are trying to use the 18S and 28S rDNA sequences to discuss the phylogeny of Thripinae in Taiwan. Sequences about 2800bp of 28S rDNA from 70 individuals of 30 species within 12 genera, and about 1800bp of 18S rDNA from 37 individuals of 28 species within 18 genera were undertaken. In 28S rDNA sequences, the uncorrected sequence divergences among individuals within species were 0–1%, and that between species within a given genus were 1–14.5%, and that among genera were 3.5–23.2%, and is more than 25% in the family level. The substitution pattern was similar between transitions and transversions, and no saturated effect was found which indicated the sequences of the two genes have phylogenetic information. The variable regions of 28S and 18S rDNA sequences were excluding in phylogenetic analysis, both Neighbor-Joining and Bayesian inferences indicated that most thrips genera are monophyly. Results from 28S rDNA revealed four distinct lineages in Thripinae; the basal one included genera of *Frankliniella, Bathrips, Ayyaria, Scirtothrips*, and *Anaphothrips*; and the advanced lineage was composed of *Thrips, Bolacothrips, Microcephalothrips*, and *Stenchaetothrips*. However, species of *Thrips* might not be a monophyletic group. Several *Thrips* species are relative to *Microcephalothrips* and *Bolacothrips*, and some with a close affinity to *Stenchaetothrips*. One of the two transient lineages is *Megalurothrips* and the other is composed of *Dichromothrips* and *Taeniothrips*. Phylogenetic inferences depict a similar tree topology in 18S rDNA sequences. Our results depicted partial resolution of Thripinae phylogeny, it will be elucidated robustly as both 18S and 28S rDNA sequences of each examined thrips were acquired simultaneously.

# Research article: Occurrence and genetic diversity of tospoviruses in Yunnan

**Published:** 2010-10-01

**Authors:** Z-k Zhang, J-H. Dong, Y-Y. Yin, X-F. Cheng, M. Ding, Q. Fang

**Affiliations:** ^1^ Biotechnology and Genetic Germplasm Institute, Yunnan Academy of Agricultural Sciences, Kunming 650223, China; ^2^ Key Faboratory of Agricultural Biotechnology of Yunnan Province, Kunming 650223, China

Tomato spotted wilt disease was first described in Sichuan province of China in 1944. The pathogen was confirmed in the mid-1980s by using ELISA and electron microscopy. In 1991, diseases caused by tospoviruses were observed on tobacco in Dali, Yunnan province. Since then, an increasing number of tospovirus diseases have been found in tomato, pepper, tobacco, potato, squash, iris, *Phalaenopsis, Cymbidium*, faba bean, onion, *Tagetes* causing significant losses. Recently, serological and nucleotide sequence data have revealed the existence of a number of tospoviruses including TSWV, GYSV, TZSV, CaCV and INSV in Yunnan province. Molecular analysis revealed that NP gene sequences of TSWV from tomato, CaCV from *Phalaenopsis* and INSV from *Phalaenopsis* have the higher identities with other isolates from different countries, respectively. The NP gene sequences of GYSV from sweet pepper had a 90.4% homology with GYSV from peanut (AF013994). TZSV is a distinct species of the genus Tospovirus isolated from tomato.

# Research article: Genetic differentiation among various populations of *Frankliniella occidentalis* (Thysanoptera: Thripidae.) assessed by mtDNA sequence and AFLP

**Published:** 2010-10-01

**Authors:** Zhi-Jun Zhang, Q.J. Wu, Y.J. Zhang, Y.B. Lu

**Affiliations:** ^1^ Institute of Vegetable and Flower, CAAS; ^2^ Institute of Plant protection and Microbiology, ZAAS, China

Since the late 1970s, western flower thrips, *Frankliniella occidentalis*, has spread from its original distribution in western North America to become a major worldwide crop pest. It was first reported in 2003, in China. This pest has established stable populations in Yunnan province and Beijing area, and a temporary population in Hangzhou, the capital of Zhejiang Province. In order to know the origin of *F. occidentalis* in China, we sequenced mtDNA COI from 103 individuals of *F. occidentalis* collected from seven regions and analysed five populations by Amplified Fragment Length Polymorphism (AFLP). From the sequencing of mtDNA COI results, six haplotypes (i.e. H1, H2, H3, H4, H5 and H6) were obtained, most of the individuals were concentrated in H1, H3 and H4, which were the worldwide ‘Greenhouse-strains’. There were four and two haplotypes in Beijing and Yunnan populations respectively. It could be assumed that there were different or multiple invasion origins of *F. occidentalis* in Beijing and Yunnan. The analysis of genetic variations among and within five populations by AFLP markers, resulted in 196 polymorphic bands, and the polymorphic rate was 88.9%, which suggested that there were significant high divergences among or within populations. The *F_st_* between populations was 0.367–0.656 and the overall *F_st_* was 0.537±0.067. Neighbourjoining analysis of populations revealed that Beijing population and the Netherlands population clustered together distinctly, which implied that the Beijing WFT population probably came from the Netherlands.
